# Linking Anthropogenic Landscape Perturbation to Herbivory and Pathogen Leaf Damage in Tropical Tree Communities

**DOI:** 10.3390/plants12223839

**Published:** 2023-11-13

**Authors:** José Luis Pablo-Rodríguez, Ángel E. Bravo-Monzón, Cristina Montiel-González, Julieta Benítez-Malvido, Sandra Álvarez-Betancourt, Oriana Ramírez-Sánchez, Ken Oyama, María Leticia Arena-Ortiz, Mariana Yólotl Alvarez-Añorve, Luis Daniel Avila-Cabadilla

**Affiliations:** 1 Laboratorio de Ecología Funcional de Sistemas Tropicales, Escuela Nacional de Estudios Superiores Unidad Mérida, Universidad Nacional Autónoma de México, Mérida 97357, Mexico; jose.pablo@enesmerida.unam.mx (J.L.P.-R.); abravomonzon@gmail.com (Á.E.B.-M.); sandra.alvarez@enesmerida.unam.mx (S.Á.-B.); anairori@gmail.com (O.R.-S.); 2 Departamento de Ciencias de la Sustentabilidad, El Colegio de la Frontera Sur, San Francisco de Campeche 24500, Mexico; cristina.montiel@ecosur.mx; 3 Laboratorio de Ecología de Hábitats Alterados, Instituto de Investigaciones en Ecosistemas y Sustentabilidad, Universidad Nacional Autónoma de México, Morelia 58190, Mexico; jbenitez@cieco.unam.mx; 4 Posgrado en Ciencias Biológicas, Unidad de Posgrado, Universidad Nacional Autónoma de México, Ciudad de México 04510, Mexico; 5 Escuela Nacional de Estudios Superiores (ENES) Unidad Morelia, Universidad Nacional Autónoma de México, Morelia 58190, Mexico; kenoyama@unam.mx; 6 Laboratorio de Ecogenómica, Facultad de Ciencias, Universidad Nacional Autónoma de México, Parque Científico y Tecnológico, Mérida 97302, Mexico; leticia.arena@ciencias.unam.mx; 7 Laboratorio de Ecología Funcional de Sistemas Tropicales, Facultad de Estudios Superiores Iztacala, Universidad Nacional Autónoma de México, Tlalnepantla de Baz 54090, Mexico

**Keywords:** anthropic disturbance, tropical humid forest, herbivory damage, plant-pathogen damage, multitrophic interactions

## Abstract

Anthropogenic disturbance of tropical humid forests leads to habitat loss, biodiversity decline, landscape fragmentation, altered nutrient cycling and carbon sequestration, soil erosion, pest/pathogen outbreaks, among others. Nevertheless, the impact of these alterations in multitrophic interactions, including host–pathogen and vector–pathogen dynamics, is still not well understood in wild plants. This study aimed to provide insights into the main drivers for the incidence of herbivory and plant pathogen damage, specifically, into how vegetation traits at the local and landscape scale modulate such interactions. For this purpose, in the tropical forest of Calakmul (Campeche, Mexico), we characterised the foliar damage caused by herbivores and pathogens in woody vegetation of 13 sampling sites representing a gradient of forest disturbance and fragmentation in an anthropogenic landscape from well preserved to highly disturbed and fragmented areas. We also evaluated how the incidence of such damage was modulated by the vegetation and landscape attributes. We found that the incidence of damage caused by larger, mobile, generalist herbivores, was more sensitive to changes in landscape configuration, while the incidence of damage caused by small and specialised herbivores with low dispersal capacity was more influenced by vegetation and landscape composition. In relation to pathogen symptoms, the herbivore-induced foliar damage seems to be the main factor related to their incidence, indicating the enormous importance of herbivorous insects in the modulation of disease dynamics across tropical vegetation, as they could be acting as vectors and/or facilitating the entry of pathogens by breaking the foliar tissue and the plant defensive barriers. The incidence of pathogen damage also responded to vegetation structure and landscape configuration; the incidence of anthracnose, black spot, and chlorosis, for example, were favoured in sites surrounded by smaller patches and a higher edge density, as well as those with a greater aggregation of semi-evergreen forest patches. Fungal pathogens were shown to be an important cause of foliar damage for many woody species. Our results indicate that an increasing transformation and fragmentation of the tropical forest of southern Mexico could reduce the degree of specialisation in plant–herbivore interactions and enhance the proliferation of generalist herbivores (chewers and scrapers) and of mobile leaf suckers, and consequently, the proliferation of some symptoms associated with fungal pathogens such as fungus black spots and anthracnose. The symptoms associated with viral and bacterial diseases and to nutrient deficiency, such as chlorosis, could also increase in the vegetation in fragmented landscapes with important consequences in the health and productivity of wild and cultivated plant species. This is a pioneering study evaluating the effect of disturbances on multitrophic interactions, offering key insights on the main drivers of the changes in herbivory interactions and incidence of plant pathogens in tropical forests.

## 1. Introduction

Anthropic disturbances triggered by agriculture, forest exploitation, and the expansion of human settlements drive changes in local habitats and landscape attributes threatening biodiversity and the ecological processes underlying ecosystem integrity [1,2,3,4]. Local- and landscape-scale habitat perturbation and the consequent changes in biotic communities and ecological processes are evident in the shifts of the herbivory levels. These shifts, highly influenced in the tropics by arthropods’ responses to environmental variation, can impact plant performance (e.g., growth and seed production), their competitive ability, and, ultimately, the overall structure and functionality of plant communities [5]. These herbivory regime shifts can also be reflected at the ecosystem level because of the herbivores’ role in nutrient cycling (e.g., in the mobilisation of nitrogen, phosphorus, and carbon into the soil), biomass production, and energy transfer to higher trophic levels [6].

In anthropogenic landscapes, herbivory patterns are influenced by both the direct effects of environmental variations on the herbivore community, which depend on species’ ecological traits such as host range and mobility, and by top–down and bottom–up controls of their communities [7,8,9,10,11,12,13]. These controls can operate simultaneously and, under some conditions, may have contrasting influences on the process [12]. The sensitivity of these controls to environmental shifts across different spatial scales, combined with the high diversity of plant and herbivore species interacting in the tropics and the wide range of study designs employed to date, might contribute to the lack of clear patterns and to knowledge gaps concerning herbivory’s reaction to landscape anthropisation.

However, to date we know that shifts in local habitat attributes and changes in available habitats at the landscape level can act as “selective forces” [14] on herbivore communities by influencing (1) the source of herbivore species and the dynamics of their populations and metapopulations; (2) competitive interactions among herbivore species; and (3) the distribution and abundance of host species, and the likelihood of herbivore–host encounters. Then, environmental variability potentially defines biotic and abiotic filters that shape herbivore community attributes, consequently affecting herbivory patterns. For instance, landscape anthropisation may lead to a reduction in plant diversity, resulting in decreased herbivore diversity and herbivory levels. Conversely, an increase in pioneering species due to disturbances, having fewer defensive resources and thus more palatable leaves, can enhance herbivory levels. Additionally, anthropogenic disturbances can result in top–down positive effects on herbivory patterns, linked to a release of herbivores from natural enemy control, caused by the reduction in abundance and diversity of specialised herbivore predators. Moreover, alterations in landscape configuration impacting connectivity influence herbivore dispersion, colonisation, and metapopulation dynamics. A decreased landscape connectivity can hinder the dispersion of certain species, favouring more mobile, generalist species, such as large chewing insects. These shifts can modify plant–herbivore networks, increasing connectivity and reducing specialisation [2,15], potentially fostering biotic homogenisation by altering the trophic structure of herbivore communities [16,17].

Under disturbances, generalist herbivores can maintain consistent damage levels to vegetation, adapting to plant species composition shifts, as they can readily switch food sources [4,18]. In contrast, specialised herbivores, such as leaf suckers and miners, may face significant reductions in their mechanical and pathogenic damages to vegetation when their primary food sources decline [19]. Notably, leaf-suckers, which host numerous plant pathogens, serve as major disease vectors. For example, insect families like Cicadellidae, Fulgoroidea, Membracidae, Aleyrodidae, Aphididae, and Psyllidae can transmit over 500 plant virus diseases [20]. Thus, anthropic disturbance can determine not only the damage caused by the different herbivore guilds but also the prevalence of insect-transmitted plant pathogens.

Although the interaction between plant communities and their pathogens (e.g., viruses, bacteria, and fungi) have profound implications for ecosystem health, they remain understudied in both natural and anthropic landscapes, apart from studies carried out in productive systems. While there is extensive research on agricultural plant pathogens and their environmental triggers [21], the dynamics between wild plant communities and their pathogens are less clear. A deeper understanding is required to predict how disturbances, and their effects on host dynamics, determine pathogen vegetation damage and its broader ecosystem consequences [22]. General observation indicates strong interdependencies between plant and pathogen community diversity patterns. On one hand, the interaction of plant pathogens with their hosts have been suggested to play a key role in maintaining and promoting plant diversity, either by preventing competitive exclusion by dominant species or by triggering a compensatory response in rare species [23]. In the tropics, in particular, fungi are one of the pathogen groups that have the most significant influence on vegetation, causing most of the pathogen-related damage [22,24,25]. On the other hand, host community diversity can modulate pathogen prevalence, with greater plant diversity potentially reducing pathogen prevalence, depending on plant–pathogen specialisation [26].

For some pathogens like viruses, the transmission depends on vector specialisation, habitat preference, and host range, where vector degree of mobility and the presence of non-permissive hosts may represent physical barriers, modulating the risk of disease spread in plant communities [20,26,27]. In general, it can be expected that fragmentation negatively affects the dispersion of certain pathogens that rely on vectors impacted by fragmentation, while the introduction of specific types of cover in the landscape (e.g., crop fields) can favour the population of certain pathogens and their vectors. Although it is recognised that the distribution and abundance of plant pathogens, their vectors, and hosts largely depend on landscape attributes (e.g., landscape composition and configuration), the precise nature of these relationships also remains unclear and understudied [28]. This is especially true in tropical anthropic landscapes.

In this study, we evaluated, at different spatial scales, the effect of anthropic changes in habitat attributes on tree community foliar damage caused by different herbivore guilds and groups of pathogens. We hypothesise that the prevalence of tree leaf damage, caused by different herbivorous guilds (e.g., gall insects, chewing insects, leaf scrapers, leaf suckers, among others) and plant pathogens (e.g., viruses, bacteria, and fungi), will be modulated by the shifts in plant community attributes (composition, taxonomic and phylogenetic diversity), vegetation structure, and landscape composition and configuration, that follow to agricultural and forestry practices. In general, we expect to find a higher level of foliar damage in vegetation stands that have undergone significant structural changes and where there has been a reduction in the richness of host species. Additionally, we expect that the reduction in the coverage of the original vegetation at the landscape scale will favour the damage caused by herbivores and pathogens with a lower degree of specialisation and a higher degree of mobility. To our knowledge, this is one of the first studies that performs a multiscale evaluation of drivers determining the prevalence of foliage damage caused by herbivorous and pathogens in a tropical anthropic landscape.

## 2. Materials and Methods

### 2.1. Study Region and Sites

**Figure 1:**
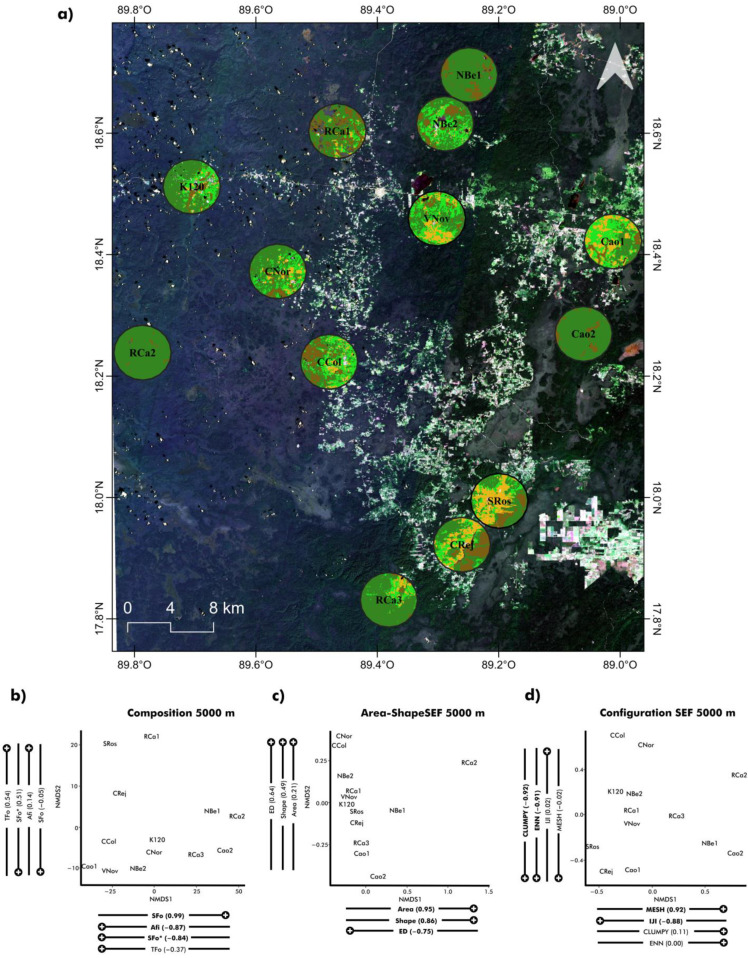
(**a**) Satellite map of the study area in the Calakmul natural reserve, circumferences illustrate 5000 m around sampling plots. Colours indicate the type of vegetation coverage at each site. (**b**) NMDS ordinations mapping landscape traits in terms of the composition of the vegetation at 5000 m around the sampling plots. (**c**) NMDS ordinations mapping landscape traits in terms of shape and area of semideciduous forest at 5000 m around sampling plots. (**d**) NMDS ordinations mapping landscape traits in terms of configuration of the vegetation at 5000 m around the sampling plots. Pearson correlation coefficients of landscape traits are located adjacently to their correspondent NDMS axes scores. Significant correlations are displayed in bold font. Nomenclature of the landscape attributes considered in ordinations: Afi (Agricultural field coverage), SFo* (Secondary semi-evergreen forest coverage), SFo (Semi-evergreen forest coverage), TFo (Thorn forest coverage), Area (Mean area of semi-evergreen forest patches coverage), ED (Edge density of semi-evergreen forest patches), Shape (Shape index of semi-evergreen forest patches), CLUMPY (Clumpiness index of patches adjacent to patches of other land-cover classes), IJI (Interspersion and juxtaposition index of the semi-evergreen forest), MESH (degree of vegetation subdivision—effective mesh size), ENN (Mean nearest neighbour distance among patches).

This study was carried out in and around the Calakmul Biosphere Reserve (CBR), located in the south of the Yucatán peninsula, in the state of Campeche, Mexico (18°36′43.2″ N, 89°32′52.8″ W) (Figure 1). The climate in this region is warm and sub-humid (Aw, Köppen classification), with a mean annual temperature ranging from 24 to 28 °C and a total annual precipitation ranging from 1000 to 1500 mm; most rainfall occurs between June and October [29,30]. The most widespread vegetation in the region is the semi-evergreen tropical forest, characterised by a tree height ranging from 18 to 25 m; approximately 25% of the vegetation sheds its leaves during the dry season. At the landscape scale, the semi-evergreen tropical forest cover is intermingled with low, flooded thorny forests, with trees ranging between 8 and 15 m in height. These forests are seasonally flooded because they are located on depressions in clay soils.

Although this region is part of the second largest extension of tropical forest in America, after the Amazon basin, and it is considered the most conserved area of continuous tropical forest in Mexico, it faces continuing threats due to expansion of agricultural fields, pastures, and government infrastructure projects, presenting an annual land-use change rate of 0.031% [30,31]. The anthropogenic disturbance coupled with other types of natural disturbance (e.g., hurricanes) result in a reduction in the extent of native vegetation and an increase in the degree of fragmentation of the remaining forest, making up a mosaic composed by secondary and old growth forest fragments of varying sizes and ages, interspersed with pastures and agricultural fields [30].

This study was carried out in thirteen permanent plots established along the study region for a long-term ecological study (Figure 1). This set of plots represents a gradient in terms of degree of preservation of semi-evergreen forest vegetation as well as in terms of the preservation of the landscape in which they are inserted. Each plot covers an area of 0.1 ha (50 × 20 m), divided in 10 quadrants of 100 m^2^ (10 × 10 m), adapting the survey method developed by Gentry [32]. All the study sites surrounding the thirteen sampling plots present the following characteristics: (1) an area of at least 100 × 100 m embedded within an area covered by the same type of vegetation, to avoid edge effect in the evaluation unit; (2) accessible by a combination of roads and trails; and (3) a minimum distance of 10,000 m between each other, minimising the likelihood of making a Type I error by the effect of pseudo-replication and spatial autocorrelation. To avoid a skewed spatial distribution, sampling sites were chosen using a balanced distribution around the reserve. This included the most representative sites of the anthropic perturbation gradient that emerges in this tropical landscape while also avoiding common statistical pitfalls associated with sub-optimal study design [33,34,35]. Google Earth high-resolution imagery (http://earth.google.com, accessed on 5 August 2019), classified images (CONABIO 2018), and the information provided by landowners, farmers, and the regional association of foresters “Productores Forestales de Calakmul A.C.” were used as well for the selection of sampling sites.

### 2.2. Vegetation Characterisation and Sampling

On each plot we identified all woody individuals with a diameter at breast height (DBH; 1.30 m) equal or greater than 5 cm. The following information was recorded for each individual: (1) species; (2) number of branches at 1.30 m; and (3) DBH. Additionally, with the help of the Forestry Pro Laser Rangerfinder (Nikon, Shanghai, China), we measured the height of the tallest tree on each of the 100 m^2^ quadrants (10 trees per plot in total), by taking three measurements per tree, which were finally averaged. We also quantified the Plant Area Index (PAI) with an LAI-2200 (LI-COR, Lincoln, NE, USA), which is the area occupied by the vegetation after being projected in a horizontal plane, and clearly reflects the variation in the number of strata [36]. Detailed information about the PAI measurement is described in Appendix A, Table A1.

For the identification of foliar damage caused by herbivores and pathogens, we sampled the most abundant species per plot (species representing at least 90% of the stand basal area or of the total woody individuals in each site), which are those that potentially have a greater impact on the ecological processes [37,38]. In total, up to three randomly selected individuals were sampled for each species per plot. In each individual, up to six leaves displaying evident damage were collected at the canopy layer with the help of a tree trimmer and highly trained tree climbers. Only fully expanded, sun-exposed, mature leaves were considered. Collected leaves were marked and placed in sealed plastic bags containing moistened paper towels and transported in a cooler to a local laboratory where they were processed.

Leaves were examined under a stereo microscope AmScope 3.5X-45X, model SM-4NTPX (United Scope LLC., Laguna Beach, CA, USA) and photographed using a digital camera model EOS Rebel T2i (CANON INC., Tokyo, Japan) to document evidence of mechanical damage from herbivores as well as the presence of pathogens (such as rust and mildew) or symptoms potentially caused by them. We focussed on diseases potentially caused by viruses, fungi, or bacteria, following descriptions of symptoms caused by plant pathogens [20,21]. We identified 11 pathological symptoms that are described in Appendix A Table A1: (1) mosaics; (2) curling leaves; (3) local lesions; (4) chlorosis; (5) necrotic patches; (6) mildew; (7) fungal spots or mycelia; (8) fungal rust; (9) anthracnosis, (10) ringspots, and (11) black non-necrotic spots.

Mechanical, non-pathogenic damages were classified into five categories corresponding to insect guilds by their feeding habits, following Andrade et al. [38]. Identifiable marks of insect herbivory are described in Appendix A, Table A1 and were divided into those caused by (1) chewing insects; (2) leaf miners; (3) leaf scrapers; (4) leaf gall insects, and (5) sap-sucking insects; this last group was divided into two subgroups: (a) mobile leaf suckers and (b) sessile leaf suckers.

### 2.3. Landscape Characterisation

The landscape surrounding the sampling plots was characterised and classified as described in Appendix A, Table A2. We used two classified satellite images from the Harmonized Landsat Sentinels-2 (HLS) project, harmonising the imagery from the NASA/USGS Landsat 8 and the ESA (European Space Agency) Sentinel-2 satellites (accessed from NASA, https://search.earthdata.nasa.gov/search, accessed on 8 September 2021). The images were classified by means of the Random Forest classification algorithms implemented in the Semi-Automatic Classification Plugin (SCP) for QGIS (http://qgis.org, accessed on 11 October 2021). During the classification process we defined Regions of Interest (ROI), as training areas for 8 land-cover classes, selected as the most conspicuous across the analysed landscape units. From these, the first four (from 1 to 4) represent the original covers of the region and the last four (from 5 to 8) correspond to covers generated as a result of human activities: (1) semi-evergreen old-growth forest; (2) thorny forest; (3) savanna; (4) water; (5) semi-evergreen secondary forest; (6) agricultural field (including growing field and pasturelands); (7) bare soil (including roads and trails); and (8) human settlements.

We characterised the landscape surrounding each study plot at three focal scales, defined by concentric circles around each plot, of 1000, 3000, and 5000 m radius (Figure 1). These focal scales allowed us to (1) encompass the home range reported for some groups of pathogens and herbivores presented in similar study systems, covering the relevant spatial scales identified in previous studies [39,40,41,42,43]; (2) avoid the spatial overlap among neighbouring buffers; and (3) identify the relevant spatial scales for those pathogens and herbivores causing leaf damage in our study system. We also characterised the landscape gradients that emerged because of human activities by means of three groups of metrics at class levels that quantified the variation in landscape composition and configuration.

First, we quantified the percentage of landscape coverage (PLAND) for the four land-cover classes that exhibited the most extensive coverage and distribution across the 13 study landscape units: semi-evergreen forest, thorny forests, semi-evergreen secondary forest, and agricultural field. These metrics might indicate the available habitat for diverse herbivore and pathogen species, given that the population dynamics of different species can be influenced by the amount of distinct vegetation types.

Second, for semi-evergreen forest patches—which represent the predominant native vegetation in the region and are the main focus of this study (given that all study plots were established within this vegetation type)—we computed the mean area (AREA), edge density (ED), and shape index (SHAPE). These indices were chosen to capture the extent of continuous habitat, the influence of edge effect (characterised by shifts in biotic and abiotic environment), and the patch shape.

Third, we calculated four indexes quantifying different aspects of landscape configuration, regarding the semi-evergreen forest: (1) Clumpiness index (CLUMPY), as a measure of the degree of patch dispersion; (2) the interspersion and juxtaposition index (IJI), as a measure of patches adjacent to patches of other land-cover classes; (3) mean nearest neighbour distance among patches (ENN_MN), as a measure of patch isolation; and (4) the effective mesh size (MESH), as a measure of the degree of vegetation subdivision. The considered landscape features can potentially affect the presence, viability, and dispersal abilities of herbivore and pathogen populations. The calculation of the landscape metrics was carried out with the Fragstat software v.4.2.1 [44,45].

### 2.4. Data Analysis

#### 2.4.1. Characterisation of Foliage Damage by Herbivorous Insects and Pathogens: Response Variables

The information generated from the inspection of the foliage was condensed into two incidence site x individual-level matrices: one recording the incidence of the different symptoms and the other one recording the incidence of the damage caused by the different insect guilds. We visualised and summarised the variation in these matrices performing non-Metric Multidimensional Scaling (NMDS) ordinations, using the “Jaccard” coefficient as a measure of dissimilarity. The determination of the number of axes for the ordinations, as well as the evaluation of their fit was made based on the resulting “stress” value, scaled from 0 to 100. A lower stress value indicates a more reliable ordination. We estimated the centroid corresponding to each site in the ordination’s spaces and tested if the distances between centroids were greater than expected by chance to evaluate differences among tree communities in the type of damage caused by herbivores and related to pathogens. We then used the centroid coordinates to evaluate for spatial autocorrelation in the type of foliage damage using Mantel tests—based on Spearman rank correlation coefficient—to test the correlation between geographic distances between sites and the centroid distances among tree communities. Significance testing (*p* ≤ 0.05) using the distance between centroids and for the Mantel test were based on 999 permutations.

We also quantified at tree individual-level per plot, the number of interactions (individual-degree) with the different types of herbivores and pathogen symptoms as a measure of the diversity of interactions between trees, insect guilds, and pathogens. Additionally, we condensed this interaction information at species level per plot and visualised the herbivore–host–symptom (pathogen) interaction networks for each tree community through multilayer networks graphs. Finally, we determined the most relevant foliage damages in each tree community by estimating the network core–peripheral index (*G_c_*) corresponding to each symptom and herbivore guild (see Appendix A, Table A3 for more details of the index).

#### 2.4.2. Parameters Summarising Vegetation and Landscape Attributes: Foliar Damage Predictors

We summarised—reducing the number of dimensions—the variation in vegetation composition and structure, and in landscape composition and configuration by performing NMDS ordinations. Through these ordinations, we generated continuous synthetic variables (axes scores), which represented explaining variables to assess foliage damage response to vegetation and landscape attributes. The ordination regarding species composition was based on a “Jaccard” dissimilarity matrix, while the rest of the ordinations were based on Euclidean distance matrices. For the analysis of the vegetation structure, we considered the following parameters: (1) number of individual trees, (2) number of branches, (3) total basal area, (4) canopy height, and (5) the plant area index. For the analyses of landscape attributes, we carried out a separate ordination for each of the three groups of landscape metrics (introduced above): (1) those quantifying the variation in landscape composition (“Composition”); (2) those quantifying the variation in the area and shape of semi-evergreen forest patches (“Area-ShapeSEF”); and (3) those quantifying the variation in the landscape configuration regarding semi-evergreen forest patches (“ConfigurationSEF”). These ordinations allowed us to deal with correlations between landscape metrics, quantifying different aspects of it, which can be correlated to each other as they are linked to the same process of habitat loss and fragmentation. A separate analysis was performed for each of the spatial scales (1000, 3000, and 5000 m radius) considered in the study (Table S1). Before carrying out the ordinations, we standardised all the parameters included in the analysis.

**Figure 2:**
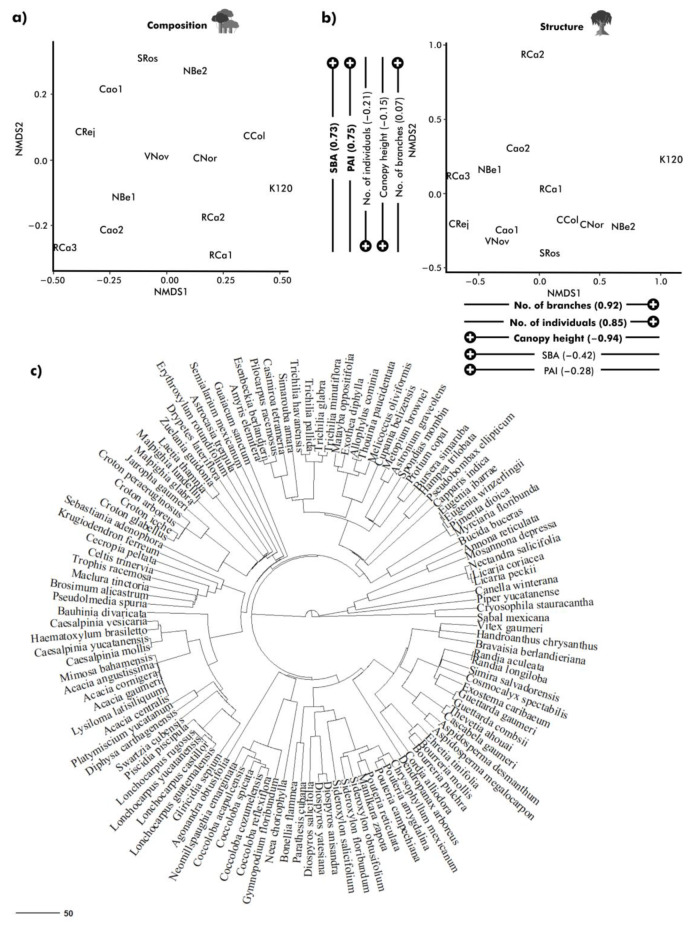
Non –Metric Multidimensional Scaling Ordinations mapping plant communities’ dissimilarities in terms of species composition (**a**) and vegetation structure (**b**). Vegetation traits considered for the ordinations were plant area index (PAI, m^2^/m^2^), total number of branches (No. branches), average of the tallest trees (Canopy Height, m), total number of individuals (No. individuals), and total basal area (SBA, m^2^/ha). Pearson correlation coefficient is presented for the structure of the vegetation describing the relationship between vegetation attributes and ordination axes. Phylogenetic tree of all species considered in this study (**c**) was calculated through the mean nearest taxonomic distance (MNTD). Branch length represent in millions of years, scalebar = 50 my.

For vegetation, we separately quantified tree species richness and the mean nearest phylogenetic distance (MNTD) for each plot. These metrics serve as measures of host species diversity and the degree of their phylogenetic relationships, respectively. For the latter, we built a phylogenetic tree including all the species recorded across the study sites by pruning the megaphylogeny provided by Qian and Jin [46], which is the largest and most up-to-date time-calibrated species-level phylogeny of seed plants. In the end, we generated an ultrametric tree with branch lengths in units of time of millions of years (Figure 2c).

#### 2.4.3. Association between Foliage Damage and the Attributes of Vegetation and Landscape

We used Phylogenetic Generalised Linear Mixed Models (PGLMMs) to test the hypothesis that variations in host community composition and diversity, vegetation structural complexity, and landscape composition and configuration were associated with variations in (1) the incidence of different types of foliage damage and (2) the tree individual degree of incidence of symptoms and interaction with insect guilds [47]. The PGLMMs allowed us to include the site-level and the overall phylogenetic-level random effects, while modelling the incidence of different types of foliage damage on tree species as a function of the environmental variation. We considered as response variables the presence–absence of different types of foliage damage (modelled with a binomial error distribution) and the tree individual degree of incidence of symptoms and interaction with insect guilds (modelled with Poisson error distribution), while as explanatory variables (fixed effects) two sets of predictors were considered: (1) all parameters related to plant community and vegetation attributes, including the synthetic variables (NMDS axes scores) summarising tree species composition and vegetation structure, as well as tree species richness and the mean nearest phylogenetic distance among host species; (2) the synthetic variables (NMDS axes scores) summarising variations in landscape composition and configuration independently for each of the considered spatial scales (1000, 3000, and 5000 m). To analyse the symptoms related to pathogens, we considered an additional set of predictors composed by the record of incidence of damage caused by the different types of herbivore guilds in individual trees. We identified predictors whose coefficients were statistically significant through hypothesis testing (*p* ≤ 0.05).

Statistical analyses were carried out in R, using the package “vegan” for the NMDS ordinations, the centroid and the Mantel tests; “bipartite” for the visualisation and analyses of interaction networks; “V.PhyloMaker’’ to generate the tree phylogeny; “picante” for the MNTD calculation; and “phyr” for the PGLMMs [48,49,50,51,52,53].

## 3. Results

### 3.1. Foliage Damage by Herbivores and Pathogens in Tree Communities in Tropical Anthropic Landscapes

A total of 2651 tree individuals, representing 118 species, 89 genera, and 43 families were identified within our study plots. The most speciose families were Fabaceae (20 species), Rubiaceae (7), Sapotaceae (7), Euphorbiaceae (6), Polygonaceae (6), and Sapindaceae (6), representing more than the 40% of the identified species. On average, 32 (range: 21–50) species and 185 (range: 111–352) individuals were present in each sampling plot (Table 1). Among the most dominant and conspicuous species—present in between 8 and 11 of the study sites—were *Bursera simaruba*, *Brosimum alicastrum*, *Lonchocarpus guatemalensis*, *Manilkara zapota*, *Krugiodendron ferreum*, and *Vitex gaumeri*.

We characterised the foliage damage in tree communities by sampling and processing 2970 leaves of 495 individuals; there were 41 individuals on average per plot (range: 26–72), representing the 84 dominant species (Tables S2 and S3) in terms of number of individuals or basal area in tree communities (see Section 2) [37,38]. All the ordinations analyses showed low stress values allowing us to properly order the study sites along the analysed environmental gradients (Figure 1 and Figure 2).

In general, the preserved forest sites (RCa2, Rca3, Nbe1, and Cao2) were similar in their landscape composition and configuration, displaying a greater and more continuous area of semi-evergreen old-growth forest (Figure 1). The sites RCa3, Cao2, and NBe1 were also very similar in their vegetation composition and structure regardless of being far from each other (Figure 2). The other nine plots (from moderately to heavily disturbed) did not show a clear pattern of grouping; on the contrary, they showed a high degree of variation in their landscape composition and configuration as well as in their vegetation composition and structure.

**Figure 3:**
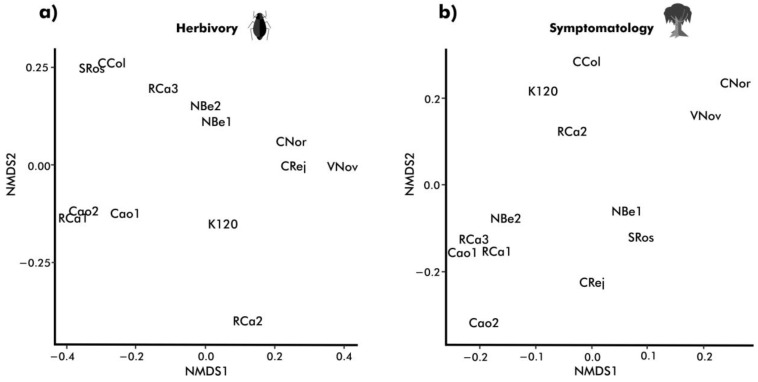
Non-Metric Multidimensional Scaling Ordinations mapping plant communities’ dissimilarities in terms of herbivory (**a**) and symptomatology (**b**).

We detected significant differences among tree communities in the composition of foliage damage caused by herbivores and related to pathogens, as shown by the resulting ordinations (NMDS) (Figure 3) with stress values of 6.67 for herbivore damage, and 11.03 for pathogen damage ordination.

The distance among the centroids corresponding to each community showed significant differences in the composition of damage caused by herbivores (r^2^ = 0.122, *p* = 0.001) and pathogens (r^2^ = 0.157, *p* = 0.001). Although the order of tree communities differs between herbivore and pathogen damage gradients, the degree of variation among communities concerning to both types of damage is quite similar, as shown by the coefficients of variation (CV) of centroids in the ordination corresponding to damage by herbivores (CV: 0.58, 0.69, and 1.04 for axis 1, axis 2, and axis 3, respectively) and damage by pathogens (0.60, 0.69, and 0.88 for axis 1, axis 2, and axis 3, respectively). No evidence of spatial structure was found in the variation of the composition of damage related to pathogens (Mantel test: r_s_ = 0.07, *p* = 0.70 for axis 1; r_s_ = 0.08, *p* = 0.25 for axis 2; r_s_ = 0.12, *p* = 0.83 for axis 3), while some signs of spatial structure were found in the case of the composition of the damage caused by herbivorous insects (Mantel test: r_s_ = 0.06, *p* = 0.32 for axis 1; r_s_ = 0.33, *p* = 0.02 for axis 2; r_s_ = 0.10, *p* = 0.22 for axis 3). The distribution of the study sites in multidimensional space in terms of type of damage did not resemble the patterns found in their landscape and vegetation structures (Figure 1 and Figure 2).

**Figure 4:**
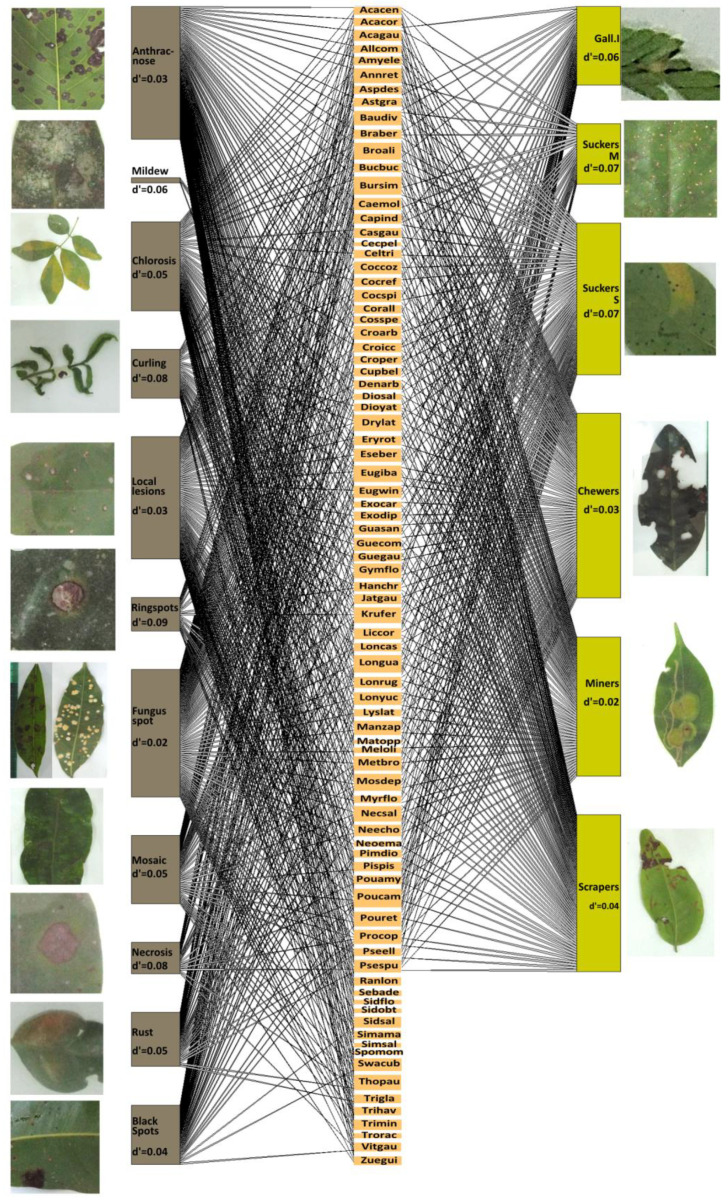
Tripartite network interaction of the tree species present at the 13 sampling plots, displaying, at the right-side, interactions with herbivore insect guilds that cause specific foliar damage and, at the left-side, the interactions with symptoms related to plant pathogens. The d index represents the degree of interaction specialisation of each foliar damage with the tree species. Each described damage is complemented by a representative image of the foliar damage found during field work. The acronyms of the species are provided in the supplementary material.

Guilds of herbivores with higher degrees of specialisation (d′ ≥ 0.06) in their interaction with tree species were—from the highest to the lowest—mobile leaf suckers, sessile leaf suckers, and leaf gall insects. Symptoms that showed a higher degree of specialisation (d′ ≥ 0.06) in their frequency among tree species were ringspots, curling leaves, necrotic patches, and mildew (Figure 4). At the community level, foliar damage caused by leaf chewers, sessile leaf sucking insects, and leaf scrapers turned out to be the most relevant (core) in the tree communities studied—in seven, three, and one communities, respectively—while the most relevant leaf symptoms were fungal spots with mycelia (in 10 communities), anthracnose (in 9 communities), and local lesions (in 7 communities) (Table 2). In the specific case of damage caused by insects, we observed the highest degree of specialisation in those tree communities with the greatest diversity of species (Figure S1).

Out of the 84 tree species analysed, 37 (44.05%) were identified as core species (Table 2). Of these, 17 species (20.24%) were identified as core in both tree interaction networks, with herbivores and with symptoms potentially associated with pathogens. Meanwhile, 11 species (13.10%) were identified as core only in the interaction with herbivores, and 9 species (10.71%) were core in the interaction with symptoms potentially associated with pathogens. The species that turned out to be core simultaneously in the herbivory and symptomatology interaction networks were those belonging to the families Burseraceae (*Bursera simaruba*, *Protium copal*), Polygonaceae (*Gymnopodium floribundum*, *Coccoloba spicata*), Sapotaceae (*Pouteria campechiana*, *Manilkara zapota*), Annonaceae (*Mosannona depressa*), Araliaceae (*Dendropanax arboreus*), Euphorbiaceae (*Croton arboreus*), Fabaceae (*Lonchocarpus guatemalensis*), Lauraceae (*Nectandra salicifolia*), Meliaceae (*Trichilia minutiflora*), Moraceae (*Brosimum alicastrum*), Nyctaginaceae (*Neea choriophylla*), Pterobryaceae (*Esenbeckia berlandieri*), Putranjivaceae (*Drypetes lateriflora*), and Sapindaceae (*Thouinia paucidentata*). Foliage of the tree species were affected, on average, by three (range: 4–2) different herbivore guilds and presented, on average, four (range: 5–3) different kinds of symptoms associated with pathogens.

### 3.2. Drivers of Mechanical Foliage Damage by Herbivores and Symptoms Related to Pathogens in Tropical Anthropogenic Landscapes

The variation in the incidence of damage caused by insect guilds was associated with changes in the composition and diversity of host communities but not with the variation in vegetation structure. In this sense, variation in gall incidence and mobile leaf sucker damage was associated with changes in the composition of tree communities (Table 3). We also found a higher incidence of galls in tree communities with a higher mean nearest phylogenetic distance. Likewise, the richness of trees showed a significant negative relationship with the incidence of damage caused by mobile leaf suckers and a marginally significant negative relationship with the damage by leaf scrapers (Table 3).

Regarding the landscape effect, the incidence of insect damage was always associated with the variation in landscape structure at the smallest spatial scale analysed (a 1000 m buffer), because the gall insects and chewers are more sensitive to changes in the landscape structure. Specifically, a higher incidence of galls and damage caused by chewers was observed in sites with a higher degree of vegetation subdivision (a low effective mesh size—MESH) and a higher degree of aggregation between patches of semi-evergreen forest (Clumpiness index—CLUMPY), which were the two attributes regarding landscape configuration that showed a higher correlation with the ordination axis that was the best predictor in these cases. Likewise, we observed a marginally significant higher incidence of damage caused by mobile leaf suckers where the semi-evergreen forest patches presented, on average, a higher area (AREA) and a lower edge density (ED). Also, we observed a higher incidence of galls in sites inserted in landscapes with a higher proportion of semi-evergreen forest and a lower proportion of agricultural fields and secondary forests (Table 3).

In general, the incidence of all the considered symptoms, except for mildew, were positively associated with the damage caused by different guilds of folivorous insects, including sessile leaf suckers and scrapers (associated with four symptoms), chewers and miners (associated with three symptoms), and mobile leaf suckers and gall insects (associated with two symptoms) (Table 4). In fact, the incidence of four of the symptoms (mosaic, necrosis, rust, and ringspot) were exclusively associated with the damage caused by folivorous insects, but not with the variation in the attributes of the vegetation and landscape. Among the symptoms associated with a greater diversity of damage by folivorous insect guilds were anthracnose and fungus spots.

However, the incidence of some symptoms associated with pathogens respond more to changes in the structure of the vegetation, which is the case for chlorosis, and black and fungus spots. The incidence of chlorosis, usually associated with nutrient deficiency and bacterial and viral diseases, is higher in secondary vegetation stands with trees or shrubs with a lower canopy height and greater number of branches. We found two patterns between symptoms associated with fungus; on one hand, the incidence of black spot was higher in vegetation stands characterised by a higher plant area index (PAI), on the other hand, the incidence of fungus spots was higher in sites with lower tree species richness and phylogenetic distance between tree species. Additionally, the incidence of local lesions, caused by viruses, was more sensitive to variation in the specific composition and species richness of tree communities, with their incidence being higher in more diverse tree communities.

In general, the incidence of pathogen damage was more associated with the variation in size and shape of semi-evergreen forest patches and their spatial distribution (Table 4). There was a higher incidence of anthracnose, chlorosis, and black spot in landscapes with smaller patches and a higher edge density (ED). Likewise, a higher incidence of anthracnose was also observed in landscapes with a lower degree of subdivision and aggregation (higher MESH and lower CLUMPY) of forest patches, while landscapes with the opposite attributes (lower MESH and higher CLUMPY) favoured a higher incidence of curling leaf symptoms, resembling the response of the damage caused by gall insects with which these symptoms are significantly related. The incidence of black spot damage was sensitive to variation in landscape structure even at larger spatial scales (e.g., 3000 and 5000 m) than those found for herbivorous insects (Table 3 and Table 4).

Finally, the average number of various types of damages caused by herbivorous and pathogens on individual trees was negatively associated with the variation in tree species richness present in the study sites (Table 3 and Table 4).

## 4. Discussion

Our highly diverse study system constituted a clear gradient of forest disturbance and fragmentation where clear patterns have emerged related to the disturbance grade of the forest. For example, the preserved forests were significantly similar in their vegetation structure and composition as well as in their landscape composition and configuration, evidencing a homogenisation of the advanced successional stages of plant communities in this region. In contrast, the disturbed forests showed great variability in all the aspects evaluated, resembling the stochastic nature of the processes operating in the early and intermediate successional stages as have been observed in other neotropical forests [54].

Across our studied gradients, changes in the structural complexity of vegetation, species richness, and the degree of relatedness between tree species, arise all together with variation in landscape composition and configuration to play different roles in the way they modulate the incidence and type of foliar damage caused by herbivores and related to pathogens. The incidence of symptoms potentially caused by pathogens was also strongly correlated with the damage caused by herbivores. This correlation was, in several cases, stronger than that between the symptoms and habitat attributes. This tight linkage might account for the similar degree of variation observed in woody communities regarding the incidence of damage caused by herbivores and symptoms associated with pathogens. However, both the patterns of these symptoms and the damage from herbivores presented distinct spatial structures, indicating that different factors are modulating them. Specifically, herbivores may depend more on their dispersal capacity along the landscape, while pathogens can rely more on their capacity to infect a plant [21].

### 4.1. Foliage Damage by Herbivores and Symptoms Related to Pathogens in Tree Communities in Tropical Anthropic Landscapes

The composition and structure of herbivore communities had a greater response to the variation in landscape and vegetation features than the communities of pathogens. Herbivores and pathogens differed in the degree of specialisation in foliage damage inflicted on vegetation (Figure S1). More specialisation in plant–herbivore interactions was usually associated with the most diverse tree communities, as a greater species richness presents a greater diversity of plant defensive strategies [55] and less optimum biotic and abiotic conditions for proliferation of generalist herbivores (e.g., stronger top–down control of herbivores by predators) [56]. In this sense, in the less diverse and more disturbed tree communities, the proliferation of generalist herbivores is expected with less specialisation in their interactions, as we found in this study.

Regarding herbivores, we found that mobile and sessile leaf suckers and leaf gall insects showed a higher degree of specialisation in their interaction with trees, as has been shown in other tropical forests [19,57,58]. The lower specialisation scores were encountered for chewer and scraper insects at most of the sites, supporting that leaf-chewing insects and leaf-scraper insects are usually generalist insect guilds [19,59,60]. Regarding pathogenic symptoms, the higher degree of specialisation in the interaction with plant species were found in those related to plant viruses such as ringspots and curling leaves.

In general, fungal pathogens have demonstrated to be an important cause of foliar damage for many woody species, representing five out of eleven symptoms reported for our plant species. Indeed, fungus spots and anthracnose were the symptoms that caused the most relevant foliar damage at most of the sites. The incidence of fungus spots was positively associated with two generalist insect guilds (chewers and scrapers) and one specialised insect guild (sessile leaf suckers). Fungal conidia responsible for anthracnose caused by *Colletotrichum* spp. has been reported to be dispersed by *Pseudotheraptus devastans*, a leaf-sucker insect able to carry and inoculate this fungal pathogen [61]. In this study, the incidence of anthracnose was positively associated with the incidence of damage by one generalist insect guild (chewer insects) and two specialised insect guilds (leaf miners and sessile leaf-sucker insects), supporting the dispersal mechanism by leaf-sucker insects.

Mildew fungal pathogens and necrotic spots were also linked to the sessile leaf suckers. The associations of these symptoms to herbivores could explain their high frequency and incidence, as some opportunistic plant fungal pathogens may take advantage of wounds inflicted by generalist herbivores [21]. Generalist herbivores were also associated with damage symptoms caused by viruses in the less diverse and most perturbed sites, as well as in their adjacent landscapes.

### 4.2. Drivers of Tree Foliage Damage by Herbivores in Tropical Anthropic Landscapes

Our results indicate that the presence of the different guilds of folivorous insects and the damage they cause to tree foliage is modulated, to a greater extent, by the variation in the composition and richness of host communities and, consequently, by the presence of susceptible tree hosts. The high susceptibility found in several herbivores to changes in host community composition can be a consequence of the high degree of specialisation that has been identified in herbivorous insects, among which there is a high proportion of family-specialist (58%) and genus-specialist (48%) species [62,63]. A high degree of specialisation is expected for endophytic insects, which were the guilds (i.e., gall and leaf-sucker insects) that in our study showed the greatest response to changes in tree community composition [62]. For galling insects, their high degree of specialisation responds to the high degree of intimacy in their interaction with its host, to be able to induce hyperplasia and hypertrophy of its tissues for the formation of galls that provide food and shelter to complete their development cycle and protect themselves against predators and environmental stressors. This kind of interaction may explain why we observed an increase in the incidence of galls in response to an increase in phylogenetic diversity in host tree communities, which may be favouring a greater diversity of galling insects associated with different lineages of trees, and consequently the overall incidence of galls in vegetation. Castagneyrol et al. [64] indicated that the phylogenetic distance is one of the main factors, along with relative abundance of host plants, that regulate specialised herbivores. Similarly, Oyama et al. [65] found in two tropical Mexican rainforests that an increase in the diversity of host species at the regional level favours an increase in gall-inducing insects. The narrow host range in gall insects can also explain why their incidence decreases when a reduction in the original vegetation occurs. Future studies should consider the potential association between changes in the phylogenetic and specific composition of host species communities and changes in the incidence and spatial distribution patterns of super-host species or taxa—those that host many gall-inducing insect species—which can greatly influence the incidence and diversity of galls in plant communities [66,67].

The negative relationship we found between the richness of tree species and the incidence of damage caused by guilds of herbivores with a high degree of specialisation such as mobile leaf suckers is consistent with the dilution effect hypothesis. The increment in tree species may result in changes in the availability of host species and the probability of encountering these species due to (1) a decrease in the population abundance of the most susceptible host species, whose distribution is possibly more patchy due to an increase in competition between tree species, or (2) an increase in the spatial association between susceptible and non-susceptible host species, where the latter can represent chemical and physical barriers disrupting olfactory recognition and colonisation of host species, consequently reducing the encounter rate between hosts and their specialised herbivore species [63,68,69].

The sensitivity and the scale at which herbivorous insects respond to variation in landscape attributes appear to be determined, in general, by their degree of affinity to different plant communities or host species, as well as their ability to disperse into different distribution patterns of remnant forest patches [70,71]. In our study, the herbivorous insects were more sensitive to variation in landscape attributes at smaller spatial scales due to the presence of physical barriers defined by the elements of the landscape. Indeed, the incidence of damage caused by insects with greater mobility capacity (e.g., chewers, mobile leaf suckers, and gall insects) showed the greatest susceptibility to variation in landscape attributes.

In the case of chewers, the greater incidence of foliar damage caused by them at sites inserted in a landscape with higher degree of vegetation subdivision may be explained by the release of predation pressure, as predators’ populations are negatively affected in fragmented landscapes [72,73]. The modification of natural forests into agricultural landscapes significantly reduces the abundance and diversity of predatory arthropods, negatively affecting their role as natural pest controllers [74]. The diversity and abundance of foliage-gleaning birds and bat communities, which can significantly reduce the overall density of arthropods (including herbivores), may also decrease because of anthropic disturbance at the landscape scale [56,75,76,77].

Moreover, chewing insects also include some of the larger size herbivorous insects (e.g., grasshoppers) with a high dispersal capacity, which facilitates their movement through the anthropic matrix, including inhospitable areas. Thus, they are able to colonise and recolonise the remnant fragments. This can be more difficult for insects of smaller size with presumably higher dispersal limitations and a higher mortality outside fragments [78]. In this sense, our results indicate that a higher degree of aggregation between the remnant fragments favours the dispersion and persistence of chewing insects. This will result in fewer local extinctions and a faster recolonisation of patches (rescue effect) [79], facilitating their metapopulation dynamics and its persistence in the system [80,81].

Our results also showed that populations of leaf-sucker insects may be more sensitive to the reduction in their natural habitats and to an increase in the edge effects, as changes in microenvironmental conditions may limit abundance and diversity of these specialised insect guilds [82]. Size and connectivity at scales higher than 300 m have been observed to impact over the population abundance and interactions of mobile leaf suckers such as the green leafhopper (*Nephottetix* spp.), brown planthopper (*Nilaparvata lugens*), and white backed planthopper (*Sogatella furcifera*) in rice fields [83]. Some functional attributes of the members of this guild may explain their sensitivity to habitat loss and fragmentation, such as a small host range, small and soft bodies, and a low dispersal distance [39,40,41].

In summary, our results indicate that the incidence of damage caused by generalist herbivorous insects with a higher degree of mobility is more sensitive to changes in landscape configuration, while the incidence of damage caused by specialist insects with low dispersal capacity is more sensitive to changes in the composition of the vegetation and the landscape.

### 4.3. Drivers of Foliage Symptoms Related to Pathogens in Tropical Anthropic Landscapes

The foliar damage caused by herbivorous insects seems to be the main factor determining the dissemination and establishment of pathogens across the foliage. This suggests that insects such as chewers, leaf suckers, leaf scrapers, miners, or galls are enormously important in the modulation of disease dynamics across tropical vegetation, as they could be acting as vectors and facilitating the entry of pathogens by breaking the foliar tissue and the plant defensive barriers. It is known for example, that certain phytopathogenic fungi are invasive opportunistic pathogens that use mechanical damage inflicted by adults and larvae herbivores that provide easy access to their substrates by removing physical barriers and allowing fungal colonisation on foliar tissue [84]. Leaf suckers, otherwise, could directly inject pathogens into leaves through their buccal apparatus [61].

A higher incidence of symptoms associated with viral and bacterial diseases, such as chlorosis, was associated with sites with a lower complexity of vegetation structure (i.e., lower densities of individual trees, sites with less strata and vegetation with lower canopy height), where harsher microenvironmental conditions (i.e., nutrient deficit, higher temperature and radiation, and low humidity) and pioneer plant species with lower defensive capacities can predominate. Chlorosis is a symptom that could be attributed to viral diseases when viruses affect the production of chlorophyll [20], or to deficiency of iron, which is important in many processes including chlorophyll biosynthesis. The deficiency of iron may in fact be observed when a reduction in bioavailability or Fe occur under alkaline soil conditions [85] such as those present in our study region.

Species richness and phylogenetic distance between species affected the incidence of fungus spots, which was greater in sites with lower tree species richness and phylogenetic diversity. With a greater phylogenetic distance between tree communities, fewer common pathogens are shared between them [86], leading to a dilution of susceptible hosts (dilution effect) [87], as has been observed in other pathogens such as the oak powdery mildew species [88,89] and the fungal root pathogen (*Heterobasidion annosum*) [90]. Likewise, local lesions (i.e., a localised hypersensitive response where host plants present damages containing viral infection) [91] were susceptible to variations in vegetation composition and species richness but showed higher incidence in more diverse tree communities. This susceptibility and low interaction specialisation score indicate that a greater number of tree species may increase possible combinations between host plants and viruses, making this kind of symptoms less specialised within the community.

The incidence of symptoms associated with fungal pathogens also responded to changes in the vegetation structure, suggesting that pathogens could be more susceptible to the microenvironmental conditions (v.gr. relative humidity, radiation, temperature) defined by this attribute, than herbivores themselves. In this sense, we observed that a greater incidence of foliar fungal disease was directly associated with a higher plant basal area (fungus spot) or higher plant area index (black spot), suggesting that a higher prevalence of these symptoms could be attributed to areas with a reduced incidence of radiation and heat, which is expected for fungi establishment and development as they are highly sensitive to temperature and environmental humidity. Microenvironmental conditions optimal for the requirements of this kind of plant pathogen have been previously reported to be found under foliage shade, which maintains the cooler temperature and high humidity [92] needed for the development of many fungal plant pathogens such as *Phytophthora infestans* [93] or *Mycospaherella graminicola* [94], providing shorter periods of incubation and increasing sporulation at lower temperatures.

### 4.4. Guild-Vector Drivers of Tree Foliage Damage by Pathogens in Tropical Anthropic Landscapes

In general, most pathogens showed a relatively weaker response to vegetation composition and diversity in contrast to the stronger response to the vegetation structure and to the composition of herbivore communities. In a similar way, the pathogen damage was more associated with the landscape configuration (i.e., variation in size and shape of forest patches and their spatial distribution) than with the landscape composition. This reinforces the idea that pathogens are highly sensitive to the quantity and spatial configuration of the semi-evergreen tropical forest, which is the original vegetation and consequently the type of vegetation evolutionarily more related to most of them. Therefore, the response of pathogens to variation in landscape attributes could be mainly determined by the response of their vectors (i.e., herbivorous insects) to these attributes as well as by their own ability to disperse and establish under the particular environmental conditions of every patch.

The incidence of anthracnose, black spots, and chlorosis was favoured in sites surrounded by smaller patches and a higher edge density, as well as with a greater aggregation of semi-evergreen forest patches. This may indicate that the edge effect and higher connectivity among patches play an important role for these diseases to be spread. In the case of chlorosis, an increase in the edge effect on the surrounding areas can enhance the possibility of viral infections to overrun plant defence mechanisms, adapting to more potential hosts. Additionally, chlorosis was associated with the variations in the incidence of damage by sessile leaf-sucking insects, which could also explain the transmission of this viral-pathogen-related symptoms [95,96]. The range of mobility of sessile leaf-sucking insects through trees is reduced and is more likely to be dependent on the size and shape of the closest forest fragments at the smallest scale (1000 m radius). In the case of black spots, greater edge zones and smaller fragments of semi-evergreen forest could promote the establishment of fungal spores related to this symptom, suggesting that factors such as speed of the wind—which may have better chances to increase at the edge areas—is an important dispersal factor for these fungal spores present at the highest analysed scale [42]. A similar effect has been observed in *Hymenoscyphus fraxineus* that causes Ash dieback disease, which has been reported to have a very efficient airborne dispersal ability in a radius of 1.5–2.5 km [97]. Also, larger fragments of original forest may reduce the population density of potential host plants, which can reduce the incidence of other fungal plant pathogens [98]. The susceptibility of black spots to the variation in leaf miners and gall insects may suggest a possible interaction of fungal pathogens associated with the presence of gall insects, which in fact showed an association very much alike to the one that black spots have with the variation in the configuration at a 1000 m buffer. The association of gall insects with the dissemination of fungal spores has been observed in other studies with species of *Cladosporium*, *Botryosphaeria*, *Fusarium*, or *Pestalotiopsis* [99]; therefore, a possible interaction between both groups is not ruled out and dispersal occurring within a buffer of 1000 m may be favoured by members of the gall insect guild.

The curling leaf symptom is also susceptible to the variations in landscape configuration and composition in a similar way as gall insects, suggesting a possible influence of gall insects in the transmission of pathogens that induce this symptom. If curly leaves are attributed to the transmission of viruses through gall insects, it could be possible that an increase in edge zones affects the abundance and diversity of gall insects [100], affecting the incidence of curly leaves as well. The aphid species of *Pemphigus* are one of the most studied groups that have been reported to be important gall-forming insects and an important threat to food crop production due to their viral transmission capacity [101]. Then, the possibility that gall insects may transmit a range of viruses inducing curly leaves in the Calakmul area is not ruled out, but further investigation is needed to determine whether viral infections are involved in this type of symptom or if another kind of foliar disease is implied.

Finally, anthracnose was associated with the presence of insect guilds that could act as vectors and may increase at the edge and disturbed areas (v.gr. chewers) [100]. Some fungal pathogens such as *Colletotrichum legendarium*, related to anthracnose in cucumber plants, have been reported to be aided in penetration by the cucumber beetle, while the fungal pathogen *Gloeosporium musarum*, cause of the anthracnose disease of *Musa balsamiana*, is also transmitted by insect members of *Hymenoptera*, during the pollination process [95]. The increase in the population of pioneer plant species in fragmented landscapes may favour the increase in the incidence of damage caused by generalist chewing insects [56] as pioneers tend to produce highly palatable leaves for insects; their leaves can present high nutritional quality and lower toughness as they invest few resources in the production of secondary metabolites for defending their tissues [102,103].

## 5. Conclusions

Our results reinforce the growing consensus that changes in composition, diversity, and structure experienced by natural forests because of anthropic disturbances affect key ecological processes such as herbivory and disease incidence. Specifically, our data suggest the following: (1) the degree of specialisation determines the response of insect–plant interactions and pathogen–plant interactions to changes in habitat and landscapes, and, consequently, the net output of such interactions; (2) the diversity of host species is one of the main drivers for the incidence of damage caused by herbivores; (3) the incidence of damage caused by larger generalist herbivorous insects is more sensitive to changes in landscape configuration, while the incidence of damage caused by small specialist insects is more sensitive to changes in the composition of the vegetation and the landscape; (4) the foliar damage caused by herbivorous insects seems to be one of the main factors related to the dissemination and establishment of pathogens across foliage, indicating the enormous importance of these guilds in the modulation of disease dynamics in the forest, as they could be acting as vectors and/or facilitating the entry of pathogens by breaking the foliar tissue and the plant defensive barriers; (5) there is a relatively weaker response of most of the pathogen-related symptoms to vegetation composition and diversity in contrast to a stronger response to vegetation structure and to the composition of herbivore communities; (6) the damage associated with pathogens was more related with the landscape configuration (the variation in the size and shape of the semi-evergreen forest patches and the way in which they are distributed in space) than with the landscape composition; (7) fungal pathogens showed to be an important cause of foliar damage for many woody species.

As the association of fungal, viral, or bacterial symptoms to any particular insect guild is expected to occur with some of the groups present at this study, further investigation is necessary to inquire into the microbiota population and their potential insect vector population to better understand specific interactions that occur between insect vectors of the pathogens present in neotropical forests. In the same way, it is urgent to perform more studies characterising the disease dynamics in tropical forests, as the information on this subject is very scarce but indispensable for the adequate management and conservation of these ecosystems.

## Figures and Tables

**Table 1:** Structural parameters of the vegetation for each of the study sites.

Study Site	Richness	Individuals	Branches	SBA	Height	PAI	Dominant Species (p_i_)
Cao1	30	138	256	24.18	17.04	5.23	*Bursera simaruba* (0.38)
Cao2	31	175	200	38.30	15.86	4.22	*Manilkara zapota* (0.18)
CCol	29	174	348	18.74	12.28	4.46	*Lonchocarpus guatemalensis* (0.50)
CNor	50	218	373	19.69	10.72	4.64	*Vitex gaumeri* (0.16)
CRej	31	111	127	33.53	19.32	6.01	*Brosimum alicastrum* (0.53)
K120	28	352	814	25.86	9.99	4.01	*Lonchocarpus guatemalensis* (0.38)
NBe1	33	149	171	38.06	17.75	4.77	*Brosimum alicastrum* (0.23)
NBe2	31	349	586	30.33	11.56	6.10	*Bursera simaruba* (0.20)
RCa1	21	134	243	19.61	12.94	3.36	*Brosimum alicastrum* (0.16)
RCa2	36	256	275	52.55	16.93	3.07	*Krugiodendron ferreum* (0.20)
RCa3	24	134	153	41.50	20.55	5.43	*Manilkara zapota* (0.34)
SRos	33	184	353	20.48	14.05	5.75	*Bursera simaruba* (0.24)
VNov	45	165	112	27.43	16.94	6.10	*Bursera simaruba* (0.30)

Parameters: species richness (Richness), total number of individuals (Individuals), number of branches (Branches), stand basal area (SBA, m^2^/ha), average canopy height (Height, m), and average plant area index (PAI, m^2^/m^2^). The dominant species in each plot and their corresponding relative abundance (p_i_) in terms of numbers of individuals is also presented.

**Table 2:** Core elements (Gc > 1) in the interaction networks between tree species and different herbivore guilds and symptoms associated with pathogens at each of the study sites.

Type of Damage		Tree Communities													No. Sites
***Guilds***	**Net**	**RCa3**	**CRej**	**NBe1**	**VNov**	**Cao1**	**Cao2**	**RCa2**	**SRos**	**RCa1**	**CCol**	**CNor**	**NBe2**	**K120**	
Chewers	H		1.70	1.24	1.52				1.08		1.31	1.40	1.53		7
SuckersS	H					1.11	1.05	1.32							3
Scrapers	H												1.07		1
***Symptoms***															
FungalS	P	1.47	1.25	1.11		1.87	1.95	1.42	1.61	1.52			1.72	1.27	10
Anthrac	P	1.47	1.47	1.88	1.62	1.14		1.57			1.42	1.19	1.18		9
LocalL	P			1.11	1.16			1.27			1.42	1.63	1.18	1.27	7
***Species***															
Manzap	H/P				1.10/1.61		/1.01	2.21/		1.02/1.37					3/3
Mosdep	H/P				1.10/		2.26/1.01	/1.08				1.51/			3/2
Broali	H/P		1.44/		1.10/1.61					1.02/					3/1
Bursim	H/P				1.10/	/1.91			/1.02				/1.44		1/3
Drylat	H/P		/1.56		1.10/					1.02/1.37					2/2
Longua	H/P				1.10/1.61			/1.08						/1.45	1/3
Necsal	H/P				1.10/				/1.73			1.51/2.42			2/2
Poucam	H/P			/1.31		1.24/	1.17/1.65								2/2
Gymflor	H/P									/1.37	2.39/			1.37/	2/1
Neecho	H/P		1.44/	1.69/				/1.08							2/1
Cocspi	H/P												2.71/1.44		1/1
Croarb	H/P							1.22/			/1.49				1/1
Denarb	H/P								1.21/1.02						1/1
Eseber	H/P							1.22/1.08							1/1
Procop	H/P				1.10/		/1.01								1/1
Thopaua	H/P							/1.83		1.02/					1/1
Trimin	H/P	/1.61		1.69/											1/1
Trigla	H					1.24			1.21						2
Acacen	H							1.22							1
Astgra	H		1.44												1
Cocref	H				2.17										1
Eryrot	H								1.21						1
Eugiba	H	1.60													1
Exodip	H						1.17								1
Guecom	H								1.21						1
Lonyuc	H											1.51			1
Swacub	H					1.24									1
Zuegui	H											1.51			1
Annret	P					1.45									1
Baudiv	P								1.02						1
Caemol	P													1.45	1
Coccoz	P										1.49				1
Krufer	P			1.90											1
Liccor	P											1.65			1
Metbro	P				1.61										1
Pouret	P		1.56												1
Psespu	P			1.31											1

Herbivore guilds (Guild): sessile leaf suckers (SuckersS), Anthracnose (Anthrac). Pathogen symptoms (Symptoms): Fungal spot (FungalS), Local lesions (LocalL). Network (Net): tree species—herbivore guilds (H), and tree species—symptoms associated with pathogens (P). The value of G_c_ corresponding to the interaction networks between herbivores and tree species (H) appears in the numerator, while the one corresponding to the interaction between tree species and pathogen symptoms (P) appears in the denominator. Tree communities are arranged in increasing order of number of vegetation structural complexity, according to the scores of the first NMDS axis. Species list—Fabaceae (6 species): *Lonchocarpus guatemalensis* (Longua), *Acacia centralis* (Acacen), *Lonchocarpus yucatanensis* (Lonyuc), *Swartzia cubensis* (Swacub), *Bauhinia divaricate* (Baudiv), *Caesalpinia mollis* (Caemol); Sapotaceae (3): *Manilkara zapota* (Manzap), *Pouteria campechiana* (Poucamp), *Pouteria reticulata* (Pouret); Polygonaceae (3): *Gymnopodium floribundum* (Gymflo), *Coccoloba spicata* (Cocspi), *Coccoloba reflexiflora* (Cocref); Anacardiaceae (2): *Astronium graveolens* (Astgra), *Metopium brownie* (Metpium); Annonaceae (2): *Mosannona depressa* (Mosdep), *Annona reticulata* (Annret); Burseraceae (2): *Bursera simaruba* (Bursim), *Protium copal* (Procop); Lauraceae (2): *Nectandra salicifolia* (Necsal), *Licaria coriacea* (Liccor); Meliaceae (2): *Trichilia minutiflora* (Trimin), *Trichilia glabra* (Trigla); Moraceae (2): *Brosimum alicastrum* (Broali), *Pseudolmedia spuria* (Psespu); Putranjivaceae (2): *Drypetes lateriflora* (Drylat), *Coccoloba cozumelensis* (Coccoz); Sapindaceae (2): *Thouinia paucidentata* (Thopau), *Exothea diphylla* (Exodip); Araliaceae (1): *Dendropanax arboreus* (Denarb); Erythroxylaceae (1): *Erythroxylum rotundifolium* (Eryrot); Euphorbiaceae (1): *Croton arboreus* (Croarb); Myrtaceae (1): *Eugenia ibarrae* (Eugiba); Nyctaginaceae (1): *Neea choriophylla* (Neecho); Rhamnaceae (1): *Krugiodendron ferreum* (Krufer); Rubiaceae (1): *Guettarda combsii*, (Guecom); Rutaceae (1): *Esenbeckia berlandieri* (Eseber); Salicaceae (1): *Zuelania Guidonia* (Zuegui). (http://www.plantsystematics.org/reveal/pbio/fam/famabbr.html, accessed on 10 August 2022).

**Table 3:** Predictors of the variations in the incidence of damage caused by different herbivore guilds.

Response Variable	Group-Level Predictors	Predictor	β	SE	Z	*p*
Gall.I	Vegetation	mntd	0.02	0.01	2.11	0.03
	Landscape_1000_	Configuration_SEF_	−2.43	1.26	−1.92	0.05
		Composition	0.02	0.01	1.77	0.07
SuckerM	Vegetation	Richness	−0.08	0.03	−2.48	0.01
		Composition_2_	2.71	1.64	1.65	0.09
	Landscape_1000_	Area-Shape_SEF_	−1.17	0.66	−1.76	0.07
Scrapers	Vegetation	Richness	−0.09	0.05	−1.70	0.09
Chewers	Landscape_1000_	Configuration_SEF_	−2.28	1.20	−1.91	0.05
Individual-degree	Vegetation	Richness	−0.02	0.01	−1.97	0.04

Response variables: incidence of foliar damage caused by leaf gall insects (Gall.I), mobile leaf suckers (SuckerM), leaf scrapers (Scrapers), chewing insects (Chewers), as well as the number of interactions of individual trees (individual-degree) with the different types of herbivores. The explanatory variables corresponding to the different groups of predictors (group-level predictors) are (1) regarding the vegetation attributes (Vegetation), the species richness (Richness), and the mean nearest phylogenetic distance among host species (mntd), and (2) regarding the landscape attributes at 1000 m scale (Landscape_1000_), the variation in the landscape composition (Composition), the area and shape of semi-evergreen forest patches (Area-Shape_SEF_), and in the landscape configuration regarding semi-evergreen forests (Configuration_SEF_). Phylogenetic Generalised Linear Mixed Models (PGLMMs) were used to model the incidence of the different types of foliage damage as a function of predictors. Model parameters: regression coefficient (β), standard error (SE), Z-score (Z), and *p*-value (*p*). More information about analyses is presented in the Section 2.

**Table 4:** Predictors of the variations in the incidence of leaf symptoms associated with pathogens.

Response Variable	Group-Level Predictors	Predictor	β	SE	Z	*p*
Anthracnose	Landscape_1000_	Area-Shape_SEF_	1.22	0.36	3.36	0.00
		Configuration_SEF_	2.24	0.80	2.81	0.00
	Herbivores	Chewers	0.48	0.21	2.23	0.03
		Miners	0.03	0.24	2.70	0.01
		Suckers_S_	0.60	0.23	2.61	0.01
Fungus spot	Vegetation	Richness	−0.12	0.02	−7.49	0.00
		mntd	−0.02	0.01	−2.45	0.01
		Structure_2_	1.04	0.42	2.49	0.01
	Herbivores	Chewers	0.79	0.24	3.27	0.00
		Scrapers	1.19	0.27	4.35	0.00
		Suckers_S_	1.59	0.25	6.29	0.00
Blackspot	Vegetation	Structure_2_	−1.67	0.90	−1.86	0.06
	Landscape_1000_	Configuration_SEF_	−2.48	1.45	−1.71	0.08
	Landscape_3000_	Area-Shape_SEF_	−1.84	0.78	−2.35	0.01
	Landscape_5000_	Area-Shape_SEF_	−1.85	0.77	−2.42	0.01
	Herbivores	Gall.I	0.82	0.37	2.23	0.03
		Miners	0.92	0.29	3.21	0.00
Local lesions	Vegetation	Composition_1_	2.94	0.87	3.40	0.00
		Richness	0.04	0.02	2.25	0.02
	Herbivores	Chewers	0.62	0.21	2.90	0.00
		Scrapers	0.53	0.24	2.24	0.03
Mosaic	Herbivores	Suckers_M_	1.19	0.41	2.87	0.00
Necrosis	Herbivores	Scrapers	0.96	0.49	1.98	0.05
		Suckers_S_	0.94	0.48	1.97	0.05
Rust	Herbivores	Miners	1.03	0.34	3.03	0.00
Ringspots	Herbivores	Scrapers	0.96	0.46	2.09	0.04
Chlorosis	Vegetation	Structure_1_	−1.78	0.96	−1.86	0.06
	Landscape_1000_	Area-Shape_SEF_	1.12	0.49	2.27	0.02
	Herbivores	Suckers_S_	0.49	0.23	2.15	0.03
Curling	Landscape_1000_	Composition	0.02	0.01	2.32	0.02
		Configuration_SEF_	−2.09	1.08	−1.93	0.05
	Landscape_3000_	Area-Shape_SEF_	−1.02	0.59	−1.71	0.08
	Herbivores	Gall.I	0.99	0.38	2.59	0.01
Individual-degree	Vegetation	Richness	−0.01	0.01	−2.14	0.03

Response variables: incidence of several symptoms associated with pathogens as well as the number of interactions of individual trees (individual-degree) with the different types of symptoms. The explanatory variables corresponding to the different groups of predictors (group-level predictors) are (1) regarding herbivore damage (Herbivores), the incidence of damage caused by chewing insects (Chewers), leaf miners (Miners), leaf scrapers (Scrapers), leaf gall insects (Gall.I), sessile leaf suckers (SuckerS), and mobile leaf suckers (SuckerM); (2) regarding the vegetation attributes (Vegetation), the species richness (Richness), the mean nearest phylogenetic distance among host species (mntd), the variation in tree community composition (Composition_1_), and the variation in vegetation structural complexity (Structure1, Structure2); and (3) regarding the landscape attributes (Landscape), the variation in the landscape composition (Composition), in the area and shape of semi-evergreen forest patches (Area-Shape_SEF_) and in the landscape configuration regarding semi-evergreen forest (Configuration_SEF_). Subscripts in vegetation attributes correspond to the ordination axis whose scores were used as synthetic variables, while in the group-level of predictors regarding landscape attributes correspond to the analysed spatial scale (1000, 3000 and 5000 m). Phylogenetic Generalised Linear Mixed Models (PGLMMs) were used to model the incidence of the different types of symptoms as a function of predictors. Model parameters: regression coefficient (β), standard error (SE), Z-score (Z), and *p*-value (*p*). More information about analyses is presented in the Section 2.

## Data Availability

Data is contained within the article or supplementary material. The data presented in this study are available in Tables S2 and S3.

## Supplementary Materials

The following supporting information can be downloaded at https://www.mdpi.com/article/10.3390/plants12223839/s1, Figure S1: Qualitative tripartite graphs (A) showing the interaction among tree species and herbivorous insect guilds (right-side) and foliar pathogens (left-side) at each study site. The degree of interaction specialisation (d′) between each guild of herbivores and pathogen-associated symptoms with the tree species is also shown. The variation in this degree of interaction specialisation (d′) regarding herbivorous guilds (B) and pathogen-associated symptoms (C) is also shown. Tree communities are arranged in decreasing order of species richness: *Acacia centralis* (Acacen), *Acacia cornigera* (Acacor), *Acacia gaumeri* (Acagau), *Allophylus cominia* (Allcom), *Amyris elemifera* (Amyele), *Annona reticulata* (Annret), *Aspidosperma desmanthum* (Aspdes), *Astronium graveolens* (Astgra), *Bauhinia divaricate* (Baudiv), *Bravaisia berlandieriana* (Braber), *Brosimum alicastrum* (Broali), *Bucida buceras* (Bucbuc), *Bursera simaruba* (Bursim), *Caesalpinia mollis* (*Caemol*), *Capparis indica* (Capind), *Cascabela gaumeri* (Casgau), *Cecropia peltate* (*Cecpel*), *Celtis trinervia* (*Celtri*), *Coccoloba cozumelensis* (Coccoz), *Coccoloba reflexiflora* (Cocref), *Coccoloba spicata* (Cocspi), *Cordia alliodora* (Corall), *Cosmocalyx spectabilis* (Cosspe), *Croton arboreus* (Croarb), *Croton icche* (Croicc), *Croton peraeruginosus* (Croper), *Cupania belizensis* (Cupbel), *Dendropanax arboreus* (Denarb), *Diospyros salicifolia* (Diosal), *Diospyros yatesiana* (Dioyat), *Drypetes lateriflora* (Drylat), *Erythroxylum rotundifolium* (Eryrot), *Esenbeckia berlandieri* (Eseber), *Eugenia ibarrae* (Eugiba), *Eugenia winzerlingii* (Eugwin), *Exostema caribaeum* (Exocar), *Exothea diphylla* (Exodip), *Guaiacum sanctum* (Guasan), *Guettarda combsii* (Guecom), *Guettarda gaumeri* (Guegau), *Gymnopodium floribundum* (Gymflo), *Hampea trilobata* (Hamtri), *Handroanthus chrysanthus* (Hanchr), *Jatropha gaumeri* (Jatgau), *Krugiodendron ferreum* (Krufer), *Licaria coriacea* (Liccor), *Lonchocarpus castilloi* (Loncas), *Lonchocarpus guatemalensis* (Longua), *Lonchocarpus rugosus* (Lonrug), *Lonchocarpus yucatanensis* (Lonyuc), *Lysiloma latisiliquum* (Lyslat), *Manilkara zapota* (Manzap), *Matayba oppositifolia* (Matopp), *Melicoccus oliviformis* (Meloli), *Metopium brownie* (Metbro), *Mosannona depressa* (Mosdep), *Myrciaria floribunda* (Myrflo), *Nectandra salicifolia* (Necsal), *Neea choriophylla* (Neecho), *Neomillspaughia emarginata* (Neoema), *Pimenta dioica* (Pimdio), *Piscidia piscipula* (Pispis), *Pouteria amygdalina* (Pouamy), *Pouteria campechiana* (Poucam), *Pouteria reticulata* (Pouret), *Protium copal* (Procop), *Pseudobombax ellipticum* (Pseell), *Pseudolmedia spuria* (Psespu), *Randia longiloba* (Ranlon), *Sebastiania adenophora* (Sebade), *Sideroxylon floribundum* (Sidflo), *Sideroxylon obtusifolium* (Sidobt), *Sideroxylon salicifolium* (Sidsal), *Simarouba amara* (Simama), *Simira salvadorensis* (Simsal), *Spondias mombin* (Spomom), *Swartzia cubensis* (Swacub), *Thouinia paucidentata* (Thopau), *Trichilia glabra* (Trigla), *Trichilia havanensis* (Trihav), *Trichilia minutiflora* (Trimin), *Trophis racemosa* (Trorac), *Vitex gaumeri* (*Vitgau*), *Zuelania guidonia* (Zuegui); Table S1: Pearson’s correlation coefficient quantifying the relationship between the landscape attributes and the first axis resulting from ordinations (NMDS) regarding different group-levels of predictors and spatial scales. Spatial scales: circular buffers of 1000, 3000, and 5000 m radius. Group-levels of predictors: variation in the landscape composition (Composition), in the area and shape of semi-evergreen forest patches (Area-Shape_SEF_), and in the landscape configuration regarding semi-evergreen forests (Configuration_SEF_). Landscape attributes: considered as part of the group-level predictor “Composition”, the percentage of landscape coverage regarding semi-evergreen forest (SFo), thorn forest (TFo), secondary semi-evergreen forest (SFo*), and agricultural field (AFi); considered as part of the group-level predictor “Area-Shape_SEF_”, the mean area (Area), shape index (Shape), and edge density (ED) of semi-evergreen forest patches; considered as part of the group-level predictor “Configuration_SEF_”, the Clumpiness index (CLUMPY), the interspersion and juxtaposition index (IJI), the mean nearest neighbour distance among patches (ENN), and the effective mesh size (MESH) regarding semi-evergreen forest patches. Significant correlations are displayed in bold font. More information about analyses is presented in the Section 2; Table S2: Incidence of damage caused by different herbivorous guilds in the species studied at the research sites; Table S3: Incidence of symptoms related to pathogens in the species studied at the research sites.

## Appendix A

**Table A1:** Methodology used for the characterisation of the vegetation on site, sampling strategy, and classification of the foliage damage.

Vegetation Characterisation and Sampling	
Characterisation of the Plant Area Index	- Plant Area Index (PAI, plant area/ground area, m^2^/m^2^) was recorded as defined in Fournier et al. [36], taking 18 points, regularly distributed every 10 m, along three 50 m transects. We recorded the PAI at each point with four measurements toward each cardinal point (twelve measurements in total), performing a reference reading every 30 min in an open space, to correct for sunlight brightness variations. We designed this sampling scheme for PAI reading seeking to avoid overlapping between adjacent measurements, considering the average height of trees, while standardising the way measurements were taken across all sites [104]. All PAI measurements were carried out during the morning (from 08:00 to 11:00 h), with a LAI-2200 Plant Canopy Analyzer (LI-COR, USA), using a viewcap of 45°.
Classification of the foliage damage in mechanical herbivory-like damage and pathogen-like damage	- Types of foliar pathogen damage related to viruses, fungi, or bacterial diseases, following descriptions of symptoms caused for plant pathogens [20,21]: (1) mosaics—changes in the amount of chlorophyl present at the foliar blade, which forms patterns of different intensities of colour; (2) curling leaves—deformation of the leaf blade by folding itself in an irregular shape, borders of the leaves are curly; (3) local lesions—localised spots of necrotic or chlorotic areas; (4) chlorosis—zones of the leaf with no chlorophyl, usually present in an irregular shape; (5) necrotic patches—big patches of leaf that are completely dead, black or brown colours are characteristic of these patches; (6) mildew—powdery spore residues in the adaxial or abaxial part of the leaf, colours of these range from white to grey or black; (7) fungal spots or mycelia—spots with different colours and shapes that display mycelia growth with a centroid, extending through the lamina; (8) fungal rust—orange to red patches of powdery spores usually present at the adaxial part of the leaf; (9) anthracnosis—dark coloured spots or sunken lesions with a slightly raised rim; (10) ringspots—changes in colour of the foliar blade in a circular ring shape; and (11) black non-necrotic spots—dark brown to black areas with different sizes and shapes, present at the foliar blade and veins. - Types of mechanical, non-pathogenic, damages identified were those caused by (1) chewing insects—any completely missing area of the foliar area, at the border or centre of the leaves; (2) leaf miners—removal of the leaf mesophyll, conserving abaxial and adaxial surface, leaving a scar in a shape of serpentine path or blotched dead areas in the leaves; (3) leaf scrapers—damage where only one part of the leaf is removed from the abaxial or adaxial part of the leaf surface, leaving the ribs and no hole is formed; (4) leaf gall insects—protuberances at the abaxial and/or adaxial side of the leaves; and (5) sap-sucking insects—regular or irregular shape scars, as well as small bumps or holes in the leaf veins or part of the blade. This last group was divided into two subgroups: (a) mobile leaf suckers—where insect bites display small bumps close to foliar veins and stems and (b) sessile leaf suckers—when scale insects were present.

**Table A2:** Description of the landscape characterisation through satellite images, using three focal scales and description of the parameters used for the characterisation of the anthropic gradient used in this work.

Landscape Characterisation	
Image classification	We used the surface reflectance S30 imagery, generated through the Sentinels-2 data and adjusted to Landsat 8 spectral response function. We looked for the following characteristics: (1) 30 m nominal spatial resolution; (2) atmospherically corrected; (3) acquired during the dry season (February 2021), due to the advantage of having a reduced number of clouds, which facilitates the discrimination of the main types of vegetation in the study region; (4) projected onto the WGS84/UTM coordinate system; and (5) constituted by the 10 spectral bands (B02: blue, B03: green, B04: red, B05: red-Edge 1, B06: red-Edge 2, B07: red-Edge 3, B08: NIR Broad, B8A: NIR Narrow, B11: SWIR 1, and B12: SWIR 2).

**Table A3:** Description of the data processing for the construction of matrices for variables of response and predictors used in this work. Additionally, description of statistical analyses and software employed are detailed.

Data Analysis	
*G_c_* index calculation	This *G_c_* index was calculated as *G_c_* = (*K_i_* − *K_mean_*)/SD_k_ × *K_i_*; where *K_i_* is the number of links (interactions) for each symptom and type of herbivore damage; *K_mean_* is the mean number of links in the network for all symptoms and types of herbivore damage; and SD_k_ is their corresponding standard deviation in the number of links [105]. The core foliage damage—those present in a significantly higher number of species—were those presenting a *G_c_* > 1, while the peripheral ones present a *G_c_* < 1.

## References

[1] Tylianakis J.M., Morris R.J. (2017). Ecological Networks Across Environmental Gradients. Annu. Rev. Ecol. Evol. Syst..

[2] Silveira L.T., de Araújo W.S. (2021). Plant-Herbivore Networks Composed by Adult and Immature Insects Have Distinct Responses to Habitat Modification in Brazilian Savannas. J. Insect Conserv..

[3] Laurance W.F., Delamônica P., Laurance S.G., Vasconcelos H.L., Lovejoy T.E. (2000). Rainforest Fragmentation Kills Big Trees. Nature.

[4] Tscharntke T., Brandl R. (2004). Plant-Insect Interactions in Fragmented Landscapes. Annu. Rev. Entomol..

[5] Crawley M.J. (1989). Insect Herbivores and Plant Population Dynamics. Annu. Rev. Entomol..

[6] Metcalfe D.B., Asner G.P., Martin R.E., Silva Espejo J.E., Huasco W.H., Farfán Amézquita F.F., Carranza-Jimenez L., Galiano Cabrera D.F., Baca L.D., Sinca F. (2014). Herbivory Makes Major Contributions to Ecosystem Carbon and Nutrient Cycling in Tropical Forests. Ecol. Lett..

[7] Scherber C., Eisenhauer N., Weisser W.W., Schmid B., Voigt W., Fischer M., Schulze E.-D., Roscher C., Weigelt A., Allan E. (2010). Bottom-up Effects of Plant Diversity on Multitrophic Interactions in a Biodiversity Experiment. Nature.

[8] De La Vega X., Grez A.A., Simonetti J.A. (2012). Is Top-down Control by Predators Driving Insect Abundance and Herbivory Rates in Fragmented Forests?. Austral Ecol..

[9] Schüepp C., Uzman D., Herzog F., Entling M.H. (2014). Habitat Isolation Affects Plant–Herbivore–Enemy Interactions on Cherry Trees. Biol. Control.

[10] Chávez-Pesqueira M., Carmona D., Suárez-Montes P., Núñez-Farfán J., Aguilar R. (2015). Synthesizing Habitat Fragmentation Effects on Plant–Antagonist Interactions in a Phylogenetic Context. Biol. Conserv..

[11] Genua L., Start D., Gilbert B. (2017). Fragment Size Affects Plant Herbivory via Predator Loss. Oikos.

[12] Rossetti M.R., Tscharntke T., Aguilar R., Batáry P. (2017). Responses of Insect Herbivores and Herbivory to Habitat Fragmentation: A Hierarchical Meta-Analysis. Ecol. Lett..

[13] Rossetti M.R., Rösch V., Videla M., Tscharntke T., Batáry P. (2019). Insect and Plant Traits Drive Local and Landscape Effects on Herbivory in Grassland Fragments. Ecosphere.

[14] Vellend M. (2010). Conceptual Synthesis in Community Ecology. Q. Rev. Biol..

[15] Oliver T.H., Morecroft M.D. (2014). Interactions between Climate Change and Land Use Change on Biodiversity: Attribution Problems, Risks, and Opportunities. Wiley Interdiscip. Rev. Clim. Chang..

[16] Thies C., Steffan-Dewenter I., Tscharntke T. (2003). Effects of Landscape Context on Herbivory and Parasitism at Different Spatial Scales. Oikos.

[17] Gossner M.M., Lewinsohn T.M., Kahl T., Grassein F., Boch S., Prati D., Birkhofer K., Renner S.C., Sikorski J., Wubet T. (2016). Land-Use Intensification Causes Multitrophic Homogenization of Grassland Communities. Nature.

[18] Tscharntke T., Steffan-Dewenter I., Kruess A., Thies C. (2002). Characteristics of Insect Populations on Habitat Fragments: A Mini Review. Ecol. Res..

[19] Novotny V., Miller S.E., Baje L., Balagawi S., Basset Y., Cizek L., Craft K.J., Dem F., Drew R.A.I., Hulcr J. (2010). Guild-Specific Patterns of Species Richness and Host Specialization in Plant–Herbivore Food Webs from a Tropical Forest. J. Anim. Ecol..

[20] Hull R. (2014). Plant Virology.

[21] Agrios G.N. (2005). Plant Pathology.

[22] García-Guzmán G., Trejo I., Sánchez-Coronado M.E. (2016). Foliar Diseases in a Seasonal Tropical Dry Forest: Impacts of Habitat Fragmentation. For. Ecol. Manag..

[23] Bradley D.J., Gilbert G.S., Martiny J.B.H. (2008). Pathogens Promote Plant Diversity through a Compensatory Response. Ecol. Lett..

[24] Viswanathan A., Ghazoul J., Honwad G., Arun Kumar N., Bagchi R. (2019). The Effects of Rainforest Fragment Area on the Strength of Plant–Pathogen Interactions. Biol. Lett..

[25] Benítez-Malvido J., Rodríguez-Alvarado G., Álvarez-Añorve M., Ávila-Cabadilla L.D., del-Val E., Lira-Noriega A., Gregorio-Cipriano R. (2021). Antagonistic Interactions Between Fusaria Species and Their Host Plants Are Influenced by Host Taxonomic Distance: A Case Study from Mexico. Front. Ecol. Evol..

[26] Liu X., Chen L., Liu M., García-Guzmán G., Gilbert G.S., Zhou S. (2020). Dilution Effect of Plant Diversity on Infectious Diseases: Latitudinal Trend and Biological Context Dependence. Oikos.

[27] Zaitlin M., Hull R. (1987). Plant Virus-Host Interactions. Annu. Rev. Plant Physiol..

[28] Plantegenest M., Le May C., Fabre F. (2007). Landscape Epidemiology of Plant Diseases. J. R. Soc. Interface.

[29] Garcia Gil G., Prieto P., Luis J., Pérez O., Arturo M. (2002). Reconocimiento Geomorfológico e Hidrográfico de La Reserva de La Biosfera Calakmul, México. Investig. Geográficas (Mx).

[30] Ramírez-Delgado J.P., Christman Z., Schmook B. (2014). Deforestation and Fragmentation of Seasonal Tropical Forests in the Southern Yucatán, Mexico (1990–2006). Geocarto Int..

[31] Ruiz-López R. (2010). Estimación y Actualización al 2009 de La Tasa de Transformación Del Hábitat de Las Áreas Naturales Protegidas SINAP I y SINAP II Del FANP: Reserva de la Biosfera Calakmul.

[32] Gentry A.H. (1982). Patterns of Neotropical Plant Species Diversity. Evol. Biol..

[33] Hurlbert S.H. (1984). Pseudoreplication and the Design of Ecological Field Experiments. Ecol. Monogr..

[34] Legendre P. (1993). Spatial Autocorrelation: Trouble or New Paradigm?. Ecology.

[35] Eigenbrod F., Hecnar S.J., Fahrig L. (2011). Sub-Optimal Study Design Has Major Impacts on Landscape-Scale Inference. Biol. Conserv..

[36] Fournier R.A., Mailly D., Walter J.-M.N., Soudani K. (2003). Remote Sensing of Forest Environments: Concepts and Case Studies.

[37] Cornelissen J.H.C.C., Lavorel S., Garnier E., Díaz S., Buchmann N., Gurvich D.E., Reich P.B., ter Steege H., Morgan H.D., van der Heijden M.G.A. (2003). A Handbook of Protocols for Standardised and Easy Measurement of Plant Functional Traits Worldwide. Aust. J. Bot..

[38] Andrade J.F., Alvarado F., Carlos Santos J., Santos B.A. (2020). Rainfall Reduction Increases Insect Herbivory in Tropical Herb Communities. J. Veg. Sci..

[39] Loxdale H.D., Hardie J., Halbert S., Foottit R., Kidd N.A.C., Carter C.I. (1993). The Relative Importance of Short- and Long-Range Movement of Flying Aphids. Biol. Rev..

[40] Thein M.M., Jamjanya T., Kobori Y., Hanboonsong Y. (2012). Dispersal of the Leafhoppers Matsumuratettix Hiroglyphicus and Yamatotettix Flavovittatus (Homoptera: Cicadellidae), Vectors of Sugarcane White Leaf Disease. Appl. Entomol. Zool..

[41] Ludwig M., Ludwig H., Conrad C., Dahms T., Meyhöfer R. (2019). Cabbage Whiteflies Colonise Brassica Vegetables Primarily from Distant, Upwind Source Habitats. Entomol. Exp. Appl..

[42] Aylor D.E. (2003). Spread of Plant Disease on a Continental Scale: Role of Aerial Dispersal of Pathogens. Ecology.

[43] West J.S. (2014). Plant Pathogen Dispersal. eLS.

[44] McGarigal K., Cushman S.A. (2002). Comparative Evaluation of Experimental Approaches to the Study of Habitat Fragmentation Effects. Ecol. Appl..

[45] Leitao A.B., Miller J., Ahern J., McGarigal K. (2006). Measuring Landscapes: A Planner’s Handbook.

[46] Qian H., Jin Y. (2016). An Updated Megaphylogeny of Plants, a Tool for Generating Plant Phylogenies and an Analysis of Phylogenetic Community Structure. J. Plant Ecol..

[47] Ives A.R., Helmus M.R. (2011). Generalized Linear Mixed Models for Phylogenetic Analyses of Community Structure. Ecol. Monogr..

[48] Dormann C.F., Gruber B., Fruend J., Fründ J. (2008). Introducing the bipartite Package: Analysing Ecological Networks. R News.

[49] Kembel S.W., Cowan P.D., Helmus M.R., Cornwell W.K., Morlon H., Ackerly D.D., Blomberg S.P., Webb C.O. (2010). Picante: R Tools for Integrating Phylogenies and Ecology. Bioinformatics.

[50] Jin Y., Qian H.V. (2019). PhyloMaker: An R Package That Can Generate Very Large Phylogenies for Vascular Plants. Ecography.

[51] Ives A., Dinnage R., Nell L.A., Helmus M., Li D. (2022). Package phyr: Model Based Phylogenetic Analysis. R Package Version 1.1.0. https://cran.r-project.org/web/packages/phyr/phyr.pdf.

[52] Oksanen J., Simpson G.L., Blanchet F.G., Kindt R., Legendre P., Minchin P.R., O’Hara R.B., Solymos P., Stevens M.H.H., Szoecs E. (2022). Package egan: Community Ecology Package. https://cran.r-project.org/web/packages/vegan/vegan.pdf.

[53] R Core Team (2022). R: A Language and Environment for Statistical Computing.

[54] Alvarez-Añorve M.Y., Quesada M., Sánchez-Azofeifa G.A., Avila-Cabadilla L.D., Gamon J.A. (2012). Functional Regeneration and Spectral Reflectance of Trees during Succession in a Highly Diverse Tropical Dry Forest Ecosystem. Am. J. Bot..

[55] Bravo-Monzón Á.E., Montiel-González C., Benítez-Malvido J., Leticia Arena-Ortíz M., Flores-Puerto I., Chiappa-Carrara X., Daniel Avila-Cabadilla L., Yolotl Alvarez-Añorve M., Dimitrakopoulos P. (2022). The Assembly of Tropical Dry Forest Tree Communities in Anthropogenic Landscapes: The Role of Chemical Defenses. Plants.

[56] Morante-Filho J.C., Arroyo-Rodríguez V., Lohbeck M., Tscharntke T., Faria D. (2016). Tropical Forest Loss and Its Multitrophic Effects on Insect Herbivory. Ecology.

[57] Kondo T., Gullan P.J., Williams D.J. (2008). Coccidology. The study of scale insects (Hemiptera: Sternorrhyncha: Coccoidea). Rev. Corpoica—Cienc. Y Tecnol. Agropecu..

[58] Miller D.G., Raman A. (2019). Host–Plant Relations of Gall-Inducing Insects. Ann. Entomol. Soc. Am..

[59] Milne M., Walter G.H., Milne J.R. (2007). Mating Behavior and Species Status of Host-Associated Populations of the Polyphagous Thrips, Frankliniella Schultzei. J. Insect Behav..

[60] Hereward J., Hutchinson J.A., McCulloch G.A., Silva R., Walter G.H. (2017). Divergence among Generalist Herbivores: The Frankliniella Schultzei Species Complex in Australia (Thysanoptera: Thripidae). Arthropod Plant Interact..

[61] Fokunang C.N., Akem C.N., Ikotun T., Dixon A.G.O., Tembe E.A. (2000). Role of the Insect Vector, Pseudotheraptus Devastans, in Cassava Anthracnose Disease Development. Eur. J. Plant Pathol..

[62] Novotny V., Basset Y. (2005). Host Specificity of Insect Herbivores in Tropical Forests. Proc. R. Soc. B Biol. Sci..

[63] Jactel H., Brockerhoff E.G. (2007). Tree Diversity Reduces Herbivory by Forest Insects. Ecol. Lett..

[64] Castagneyrol B., Jactel H., Vacher C., Brockerhoff E.G., Koricheva J. (2014). Effects of Plant Phylogenetic Diversity on Herbivory Depend on Herbivore Specialization. J. Appl. Ecol..

[65] Oyama K., Pérez-Pérez M.A., Cuevas-Reyes P., Luna-Reyes R. (2003). Regional and Local Species Richness of Gall-Inducing Insects in Two Tropical Rain Forests in Mexico. J. Trop. Ecol..

[66] Mendonça M. (2007). Plant Diversity and Galling Arthropod Diversity—Searching for Taxonomic Patterns in an Animal-Plant Interaction in the Neotropics. Bol. Soc. Argent..

[67] Santos M.G., Hanson P., Maia V.C., Mehltreter K. (2019). A Review of Galls on Ferns and Lycophytes. Environ. Entomol..

[68] Ostfeld R.S., Keesing F. (2012). Effects of Host Diversity on Infectious Disease. Annu. Rev. Ecol. Evol. Syst..

[69] Civitello D.J., Cohen J., Fatima H., Halstead N.T., Liriano J., McMahon T.A., Ortega C.N., Sauer E.L., Sehgal T., Young S. (2015). Biodiversity Inhibits Parasites: Broad Evidence for the Dilution Effect. Proc. Natl. Acad. Sci. USA.

[70] Habel J.C., Schmitt T. (2018). Vanishing of the Common Species: Empty Habitats and the Role of Genetic Diversity. Biol. Conserv..

[71] Gámez-Virués S., Perović D.J., Gossner M.M., Börschig C., Blüthgen N., De Jong H., Simons N.K., Klein A.M., Krauss J., Maier G. (2015). Landscape Simplification Filters Species Traits and Drives Biotic Homogenization. Nat. Commun..

[72] Kalka M.B., Smith A.R., Kalko E.K.V. (2008). Bats Limit Arthropods and Herbivory in a Tropical Forest. Science.

[73] Karp D.S., Daily G.C. (2014). Cascading Effects of Insectivorous Birds and Bats in Tropical Coffee Plantations. Ecology.

[74] Rusch A., Chaplin-Kramer R., Gardiner M.M., Hawro V., Holland J., Landis D., Thies C., Tscharntke T., Weisser W.W., Winqvist C. (2016). Agricultural Landscape Simplification Reduces Natural Pest Control: A Quantitative Synthesis. Agric. Ecosyst. Environ..

[75] Fenton M.B., Acharya L., Audet D., Hickey M.B.C., Merriman C., Obrist M.K., Syme D.M., Adkins B. (1992). Phyllostomid Bats (Chiroptera: Phyllostomidae) as Indicators of Habitat Disruption in the Neotropics. Biotropica.

[76] Medellín R.A., Equihua M., Amin M.A. (2000). Bat Diversity and Abundance as Indicators of Disturbance in Neotropical Rainforests. Conserv. Biol..

[77] Williams-Guillén K., Perfecto I., Vandermeer J. (2008). Bats Limit Insects in a Neotropical Agroforestry System. Science.

[78] Bommarco R., Biesmeijer J.C., Meyer B., Potts S.G., Pöyry J., Roberts S.P.M., Steffan-Dewenter I., Ockinger E. (2010). Dispersal Capacity and Diet Breadth Modify the Response of Wild Bees to Habitat Loss. Proc. R. Soc. B Biol. Sci..

[79] Brown J.H., Kodric-Brown A. (1977). Turnover Rates in Insular Biogeography: Effect of Immigration on Extinction. Ecology.

[80] Jonsen I.D., Fahrig L. (1997). Response of Generalist and Specialist Insect Herbivores to Landscape Spatial Structure. Landsc. Ecol..

[81] Gutiérrez D. (2002). Metapoblaciones: Un Pilar Básico En Biología de Conservación. Ecosistemas.

[82] Bagchi R., Brown L.M., Elphick C.S., Wagner D.L., Singer M.S. (2018). Anthropogenic Fragmentation of Landscapes: Mechanisms for Eroding the Specificity of Plant–Herbivore Interactions. Oecologia.

[83] Dominik C., Seppelt R., Horgan F.G., Settele J., Václavík T. (2018). Landscape Composition, Configuration, and Trophic Interactions Shape Arthropod Communities in Rice Agroecosystems. J. Appl. Ecol..

[84] Gossner M.M., Beenken L., Arend K., Begerow D., Peršoh D. (2021). Insect Herbivory Facilitates the Establishment of an Invasive Plant Pathogen. ISME Commun..

[85] Santos C.S., Ozgur R., Uzilday B., Turkan I., Roriz M., Rangel A.O.S.S., Carvalho S.M.P., Vasconcelos M.W. (2019). Understanding the Role of the Antioxidant System and the Tetrapyrrole Cycle in Iron Deficiency Chlorosis. Plants.

[86] Parker I.M., Saunders M., Bontrager M., Weitz A.P., Hendricks R., Magarey R., Suiter K., Gilbert G.S. (2015). Phylogenetic Structure and Host Abundance Drive Disease Pressure in Communities. Nature.

[87] Nguyen D., Castagneyrol B., Bruelheide H., Bussotti F., Guyot V., Jactel H., Jaroszewicz B., Valladares F., Stenlid J., Boberg J. (2016). Fungal Disease Incidence along Tree Diversity Gradients Depends on Latitude in European Forests. Ecol. Evol..

[88] Hantsch L., Braun U., Scherer-Lorenzen M., Bruelheide H. (2013). Species Richness and Species Identity Effects on Occurrence of Foliar Fungal Pathogens in a Tree Diversity Experiment. Ecosphere.

[89] Dillen M., Smit C., Buyse M., Höfte M., De Clercq P., Verheyen K. (2017). Stronger Diversity Effects with Increased Environmental Stress: A Study of Multitrophic Interactions between Oak, Powdery Mildew and Ladybirds. PLoS ONE.

[90] Piri T., Korhonen K., Sairanen A. (1990). Occurrence of *Heterobasidion annosum* in Pure and Mixed Spruce Stands in Southern Finland. Scand. J. For. Res..

[91] Loebenstein G. (2009). Local Lesions and Induced Resistance. Adv. Virus Res..

[92] Rosenvald R., Drenkhan R., Riit T., Lõhmus A. (2015). Towards Silvicultural Mitigation of the European Ash (Fraxinus Excelsior) Dieback: The Importance of Acclimated Trees in Retention Forestry. Can. J. For. Res..

[93] Maziero J.M.N., Maffia L.A., Mizubuti E.S.G. (2009). Effects of Temperature on Events in the Infection Cycle of Two Clonal Lineages of Phytophthora Infestans Causing Late Blight on Tomato and Potato in Brazil. Plant Dis..

[94] Lovell D.J., Hunter T., Powers S.J., Parker S.R., Van Den Bosch F. (2004). Effect of Temperature on Latent Period of Septoria Leaf Blotch on Winter Wheat under Outdoor Conditions. Plant Pathol..

[95] Agrios G.N., Capinera J.L. (2008). Transmission of Plant Diseases by Insects. Encyclopedia of Entomology.

[96] Herrbach E., Le Maguet J., Hommay G. (2016). CHAPTER 11: Virus Transmission by Mealybugs and Soft Scales (Hemiptera: Coccoidea). Vector-Mediated Transmission of Plant Pathogens.

[97] Grosdidier M., Ioos R., Husson C., Cael O., Scordia T., Marçais B. (2018). Tracking the Invasion: Dispersal of Hymenoscyphus Fraxineus Airborne Inoculum at Different Scales. FEMS Microbiol. Ecol..

[98] Grosdidier M., Scordia T., Ioos R., Marçais B. (2020). Landscape Epidemiology of Ash Dieback. J. Ecol..

[99] Raman A., Suryanarayanan T.S. (2017). Fungus–Plant Interaction Influences Plant-Feeding Insects. Fungal Ecol..

[100] Wirth R., Meyer S.T., Leal I.R., Tabarelli M. (2008). Plant Herbivore Interactions at the Forest Edge.

[101] Hałaj R., Osiadacz B. (2013). European Gall-Forming Pemphigus (Aphidoidea: Eriosomatidae). Zool. Anz..

[102] Coley P.D., Barone J.A. (1996). Herbivory and Plant Defenses in Tropical Forests. Annu. Rev. Ecol. Syst..

[103] Wright I.J., Reich P.B., Westoby M., Ackerly D.D., Baruch Z., Bongers F., Cavender-Bares J., Chapin T., Cornellssen J.H.C., Diemer M. (2004). The Worldwide Leaf Economics Spectrum. Nature.

[104] Nassar J.M., Rodriguez J.P., Sánchez-Azofeifa A., Garvin T., Quesada M. (2008). Manual of Methods: Human, Ecological and Biophysical Dimensions of Tropical Dry Forests.

[105] Dáttilo W., Guimarães P.R., Izzo T.J. (2013). Spatial Structure of Ant–Plant Mutualistic Networks. Oikos.

