# Supplementary material for: Linking Anthropogenic Landscape Perturbation to Herbivory and Pathogen Leaf Damage in Tropical Tree Communities
Source: Plants (Basel). 2023 Nov 13;12(22):3839. doi: 10.3390/plants12223839 (PMC10675074; doi:10.3390/plants12223839)
Supplement: Supplementary file 1 [file plants-12-03839-s001.zip › Table S1 Correlation between landscape attributes and ordinations axis.pdf]

Table S1. Pearson's correlation coefficient quantifying the relationship between the landscape attributes and the first axis resulting from ordinations (NMDS) regarding different group-level of predictors and spatial scales.

	**Spatial scale**		
	**1000 m**	**3000 m**	**5000 m**
**Attributes**		**Composition**	
SFo	**1.00**	**0.99**	**-0.84**
TFo	-0.04	-0.21	-0.37
SFo*	**-0.75**	**-0.77**	**0.99**
AFi	**-0.75**	**-0.72**	**-0.87**
		**Area-Shape$_{SEF}$**	
Area	**-0.85**	**0.59**	**0.95**
ED	**0.86**	**-0.75**	**0.86**
Shape	**0.62**	0.20	**-0.76**
		**Configuration$_{SEF}$**	
CLUMPY	**-0.78**	0.37	0.11
IJI	-0.38	**-0.89**	**-0.88**
MESH	**0.91**	**0.95**	**0.92**
ENN	-0.43	**-0.62**	0.00

Spatial scales: circular buffers of 1000, 3000 and 5000 m radius. Group-level of predictors: variation in the landscape composition (Composition), in the area and shape of semi-evergreen forest patches (Area-Shape$_{SEF}$) and in the landscape configuration regarding semi-evergreen (Configuration$_{SEF}$). Landscape attributes: considered as part of the group-level predictor "Composition", the percentage of landscape coverage regarding semi-evergreen forest (SFo), thorn forest (TFo), secondary semi-evergreen forest (SFo*), and agricultural field (AFi); considered as part of the group-level predictor "Area-Shape$_{SEF}$", the mean area (Area), shape index (Shape), edge density (ED) of semi-evergreen forest patches; and considered as part of the group-level predictor "Configuration$_{SEF}$", the clumpiness index (CLUMPY), the interspersion and juxtaposition index (IJI), the mean nearest neighbour distance among patches (ENN) and the effective mesh size (MESH) regarding semi-evergreen forest patches. Significant correlations are displayed in bold font. More information about analyses are presented in the method section.
