# Supplementary material for: Linking Anthropogenic Landscape Perturbation to Herbivory and Pathogen Leaf Damage in Tropical Tree Communities
Source: Plants (Basel). 2023 Nov 13;12(22):3839. doi: 10.3390/plants12223839 (PMC10675074; doi:10.3390/plants12223839)
Supplement: Supplementary file 1 [file plants-12-03839-s001.zip › Table S3 Incidence of symptoms related to pathogens in the studied species.pdf]

Table S3. Incidence of symptoms related to pathogens in the species studied at the research sites.

This data frame serves as the basis for various analyses, including interaction webs, non-metric multidimensional scaling (NMDS), and Generalized Linear Mixed Model (GLMM) analyses, all of which are aimed at understanding the distribution and incidence of plant symptoms related to pathogens among different species in our study.

Plot	Species	Antracnose	Mildew	Chlorosis	Curling	Local lesions	Ringspots	Fungus spot	Mosaic	Necrosis	Rust	Black spots
Cao1	Acacor	1	0	1	0	0	0	1	0	0	1	0
Cao1	Allcom	0	0	1	0	0	0	1	0	0	0	0
Cao1	Annret	1	0	1	0	1	1	1	1	0	1	1
Cao1	Bursim	1	0	1	1	1	1	1	1	1	1	0
Cao1	Eugiba	1	0	0	0	1	1	1	0	0	1	1
Cao1	Eugwin	0	0	0	0	0	0	1	1	0	0	0
Cao1	Guecom	1	0	0	0	1	0	1	0	0	1	0
Cao1	Loncas	1	0	0	0	1	0	1	1	0	0	0
Cao1	Lonrug	1	0	1	1	1	0	1	0	0	1	0
Cao1	Manzap	0	0	1	0	0	1	1	0	0	0	0
Cao1	Necsal	1	0	1	0	0	0	1	0	1	1	0
Cao1	Poucam	1	0	0	1	1	0	1	0	0	1	1
Cao1	Swacub	1	0	1	0	1	1	1	1	0	0	1
Cao1	Trigla	1	0	0	0	1	0	1	1	0	1	0
Cao1	Trorac	1	0	0	0	0	0	1	0	0	0	0
Cao2	Broali	1	0	0	1	0	1	1	0	0	1	0
Cao2	Drylat	0	0	1	0	1	0	1	0	0	0	0
Cao2	Eugiba	1	0	0	1	0	0	1	0	0	1	0
Cao2	Exodip	1	0	0	1	1	0	1	0	0	0	0
Cao2	Manzap	0	0	1	1	1	1	1	0	0	1	0
Cao2	Metbro	0	0	0	0	1	0	1	0	0	1	1
Cao2	Mosdep	1	0	0	1	0	0	1	1	0	1	1
Cao2	Pimdio	0	0	0	0	1	0	1	0	0	1	1

Cao2	Pouamy	0	0	0	0	0	0	1	0	0	0	0
Cao2	Poucam	1	0	0	1	1	1	1	0	0	1	1
Cao2	Procop	1	0	1	0	1	0	1	1	0	1	0
Cao2	Pseell	0	0	0	0	0	0	1	1	0	1	0
Cao2	Psespu	1	0	0	0	1	0	1	0	1	1	0
Cao2	Simama	1	0	1	0	1	0	1	0	0	0	0
CCol	Acagau	1	0	1	0	1	0	1	0	0	0	0
CCol	Coccoz	1	0	0	1	1	0	1	1	0	1	1
CCol	Croarb	1	0	1	0	1	0	1	0	1	1	1
CCol	Eugwin	1	0	1	0	1	0	1	0	0	0	1
CCol	Gymflo	1	0	0	0	1	0	1	0	1	0	0
CCol	Longua	1	0	0	1	1	0	0	0	1	0	1
CCol	Lyslat	1	0	0	0	1	0	0	0	0	0	0
CCol	Necsal	1	0	0	0	1	0	1	0	1	0	1
CCol	Thopau	1	0	1	0	1	0	1	1	0	0	0
CCol	Vitgau	1	0	0	0	1	0	1	0	1	0	0
CNor	Acacen	0	0	1	0	0	0	0	0	0	0	0
CNor	Acagau	0	0	1	0	1	0	0	0	0	0	0
CNor	Broali	1	0	1	0	1	0	0	0	0	0	0
CNor	Bursim	1	0	1	0	1	0	0	0	0	0	0
CNor	Casgau	0	0	1	0	1	0	1	1	0	0	1
CNor	Cocref	1	0	1	0	1	0	1	0	0	0	0
CNor	Corall	1	0	0	0	1	0	1	0	0	0	1
CNor	Croicc	1	0	1	0	1	0	1	0	0	1	0
CNor	Diosal	0	0	1	0	1	0	0	1	0	0	0
CNor	Drylat	1	0	0	0	1	0	1	0	0	0	1
CNor	Eugiba	1	0	0	1	1	0	1	0	0	1	0
CNor	Guecom	0	0	1	1	1	0	1	0	0	0	1
CNor	Guegau	1	0	0	0	1	0	0	0	0	0	0

CNor	Gymflo	1	0	1	0	1	0	0	0	0	0	0
CNor	Liccor	1	0	1	1	1	0	0	0	1	0	1
CNor	Longua	1	0	1	1	1	0	0	0	0	0	0
CNor	Lonyuc	1	0	1	0	1	0	1	1	0	0	0
CNor	Manzap	1	0	0	1	1	0	0	0	0	0	1
CNor	Metbro	1	0	1	0	1	0	0	0	0	0	0
CNor	Mosdep	1	0	1	0	1	0	1	1	0	0	0
CNor	Necsal	1	0	1	0	1	0	1	1	0	1	1
CNor	Neoema	1	0	0	0	1	0	1	0	0	0	0
CNor	Pispis	1	0	1	1	1	0	0	0	0	0	0
CNor	Sebade	1	0	1	0	1	0	0	0	0	0	0
CNor	Thopau	1	0	1	0	1	0	1	1	0	0	0
CNor	Trihav	1	0	0	0	1	0	1	1	0	0	0
CNor	Vitgau	1	0	0	0	1	0	0	0	0	0	1
CNor	Zuegui	1	0	0	0	1	0	1	0	0	0	0
CRej	Allcom	1	0	1	0	0	0	1	1	0	0	0
CRej	Annret	1	0	0	0	0	0	1	0	0	0	0
CRej	Astgra	1	0	1	1	1	0	1	0	0	0	0
CRej	Broali	1	0	1	0	0	0	1	0	0	0	1
CRej	Celtri	1	0	1	0	0	0	1	0	0	0	0
CRej	Diosal	0	0	0	0	0	0	0	1	0	0	0
CRej	Drylat	1	0	1	0	0	0	1	1	0	1	1
CRej	Krufer	1	0	0	0	0	0	1	0	0	0	0
CRej	Meloli	1	0	1	0	0	0	1	0	0	0	0
CRej	Neecho	1	0	1	0	1	0	1	1	0	0	0
CRej	Pouret	1	0	1	0	1	0	1	1	0	0	1
CRej	Simama	1	0	1	0	0	0	1	0	0	0	0
CRej	Vitgau	1	0	0	0	0	0	1	0	0	0	0
CRej	Zuegui	1	0	0	1	1	0	0	1	0	0	0

K120	Bursim	0	0	0	0	1	0	1	1	1	0	0
K120	Caemol	1	0	0	0	1	0	1	1	1	1	0
K120	Croarb	1	0	0	0	1	0	1	0	1	1	0
K120	Croper	0	0	0	0	1	0	1	0	0	1	1
K120	Eseber	1	0	0	0	1	0	1	0	0	0	1
K120	Gymflo	1	0	0	0	1	0	1	0	1	0	1
K120	Longua	1	0	0	0	1	0	1	1	1	1	0
K120	Lonyuc	1	0	0	0	1	0	1	1	0	0	1
K120	Neoema	1	0	0	0	1	0	1	0	0	0	0
K120	Sidsal	1	0	0	0	1	0	1	0	0	0	1
NBe1	Baudiv	1	0	0	0	0	1	1	0	1	0	1
NBe1	Broali	1	0	1	0	0	0	1	0	0	1	0
NBe1	Bursim	1	0	0	0	1	0	0	0	0	0	0
NBe1	Cosspe	1	0	0	0	0	0	1	0	0	0	0
NBe1	Krufer	1	0	0	1	1	1	1	0	0	1	1
NBe1	Loncas	1	0	0	0	1	0	0	1	0	0	0
NBe1	Mosdep	1	0	0	1	0	0	0	0	0	0	0
NBe1	Necsal	1	0	0	0	1	0	1	1	0	0	0
NBe1	Neecho	1	0	1	0	1	0	1	0	0	0	0
NBe1	Poucam	1	0	1	0	1	0	1	1	0	0	1
NBe1	Pouret	1	0	0	1	1	0	1	0	0	0	1
NBe1	Procop	1	0	0	0	1	0	1	0	1	0	1
NBe1	Pseell	0	0	0	0	1	0	0	0	0	0	0
NBe1	Psespu	1	0	1	1	1	0	1	0	0	1	0
NBe1	Ranlon	1	0	1	0	1	1	1	0	0	0	0
NBe1	Sidflo	1	0	0	0	1	0	0	0	0	0	0
NBe1	Sidobt	1	0	0	0	0	0	1	0	0	0	0
NBe1	Sidsal	1	0	1	0	0	0	1	0	0	0	0
NBe1	Trimin	0	0	1	1	1	0	0	1	0	0	0

NBe2	Bursim	1	0	1	1	1	0	1	1	0	0	0
NBe2	Casgau	1	0	0	0	1	0	1	0	0	0	0
NBe2	Cocspi	1	0	0	1	1	1	1	0	1	0	0
NBe2	Croarb	1	0	1	0	0	0	1	0	0	1	1
NBe2	Denarb	0	0	0	0	1	0	1	1	0	0	0
NBe2	Guecom	1	0	0	0	1	0	1	0	0	1	1
NBe2	Longua	1	0	0	0	0	0	1	0	0	1	0
NBe2	Lyslat	0	0	0	0	1	0	1	0	0	0	1
NBe2	Necsal	1	0	0	0	0	0	1	1	0	1	0
NBe2	Poucam	1	0	1	0	1	0	1	0	0	0	0
NBe2	Thopau	1	0	0	0	1	0	1	0	0	1	0
NBe2	Vitgau	0	0	0	0	1	0	0	0	0	0	0
RCa1	Acagau	0	0	1	1	0	0	0	0	0	1	0
RCa1	Broali	1	0	1	0	1	0	1	0	0	1	0
RCa1	Casgau	1	0	1	0	1	0	1	0	0	0	0
RCa1	Drylat	1	1	0	0	1	0	1	1	0	1	1
RCa1	Eryrot	0	0	0	0	1	1	1	0	0	0	0
RCa1	Eugwin	1	0	1	0	1	0	1	1	0	0	1
RCa1	Gymflo	1	0	1	1	1	0	1	1	0	0	1
RCa1	Krufer	1	0	0	0	1	0	1	0	0	1	1
RCa1	Lonyuc	1	0	1	0	0	0	1	0	0	1	0
RCa1	Manzap	1	0	1	1	1	1	1	0	0	1	0
RCa1	Mosdep	0	0	0	0	1	0	1	1	0	1	0
RCa1	Thopau	1	0	1	0	0	0	1	0	0	0	0
RCa2	Acacen	1	0	0	0	1	0	1	0	0	0	0
RCa2	Amyele	1	0	0	0	1	0	1	0	1	0	0
RCa2	Broali	1	0	1	0	0	0	1	0	0	0	0
RCa2	Capind	1	0	0	0	1	0	1	1	0	1	0
RCa2	Croarb	1	0	0	0	1	0	1	0	0	1	0

RCa2	Drylat	1	0	0	1	1	0	0	1	0	0	0
RCa2	Eseber	1	1	1	0	1	0	1	1	0	0	0
RCa2	Eugiba	1	0	1	0	1	0	1	1	0	0	0
RCa2	Eugwin	1	0	0	0	1	0	1	0	0	0	0
RCa2	Exodip	1	0	0	0	1	0	1	0	0	0	1
RCa2	Guasan	1	0	0	1	1	0	1	1	0	0	0
RCa2	Krufer	1	0	1	0	1	0	1	0	1	0	0
RCa2	Longua	1	1	1	0	1	0	1	1	0	0	0
RCa2	Lonyuc	1	0	0	0	0	0	1	0	0	0	0
RCa2	Manzap	1	0	1	0	1	0	1	0	0	0	0
RCa2	Mosdep	1	0	1	1	1	0	1	1	0	0	0
RCa2	Neecho	1	0	1	0	1	0	1	1	0	1	0
RCa2	Thopau	1	0	1	0	1	1	1	1	1	0	0
RCa3	Aspdes	0	0	1	0	0	1	1	0	0	0	0
RCa3	Eugiba	1	0	1	0	1	0	1	1	0	0	0
RCa3	Exocar	1	0	1	0	0	0	1	0	0	0	0
RCa3	Hanchr	1	0	1	0	1	1	1	0	0	0	0
RCa3	Manzap	1	0	0	1	0	0	1	0	0	0	1
RCa3	Mosdep	1	0	1	0	0	1	1	0	0	0	1
RCa3	Myrflo	1	0	0	0	1	1	1	0	0	0	0
RCa3	Pouamy	1	0	1	0	0	1	1	0	1	0	0
RCa3	Poucam	1	0	0	0	0	1	1	0	1	0	1
RCa3	Pouret	1	0	1	0	0	1	1	0	0	0	0
RCa3	Psespu	1	0	0	0	1	0	1	0	0	0	0
RCa3	Trimin	1	0	1	1	1	0	1	1	0	0	0
RCa3	Vitgau	1	0	0	0	1	0	0	0	0	0	0
SRos	Baudiv	1	0	0	1	1	0	1	1	0	0	0
SRos	Bursim	1	0	1	0	1	0	1	0	0	0	1
SRos	Cupbel	1	0	0	0	1	0	1	0	0	1	0

SRos	Denarb	1	0	1	0	1	0	1	1	0	0	0
SRos	Dioyat	1	0	0	1	0	0	1	0	0	0	1
SRos	Drylat	0	0	1	0	0	0	1	0	0	1	0
SRos	Eryrot	0	0	1	0	1	0	1	1	0	0	0
SRos	Guecom	0	0	0	0	1	0	1	0	0	0	0
SRos	Longua	1	0	1	0	1	0	1	0	0	0	0
SRos	Mosdep	1	0	1	0	0	0	1	0	0	0	0
SRos	Necsal	1	0	1	0	0	0	1	1	0	1	1
SRos	Pispis	1	0	0	1	1	0	0	0	0	0	0
SRos	Sidsal	1	0	1	0	0	0	1	1	0	0	0
SRos	Spomom	0	0	0	0	1	0	0	0	0	0	0
SRos	Swacub	1	0	0	0	1	0	1	0	0	0	0
SRos	Trigla	0	0	0	0	0	0	1	0	0	0	0
VNov	Acacen	0	0	1	0	0	0	0	0	0	0	0
VNov	Acagau	1	0	1	0	1	0	1	0	0	0	0
VNov	Allcom	0	0	0	0	0	0	1	0	0	0	0
VNov	Braber	1	0	1	0	1	0	1	1	0	0	0
VNov	Broali	1	0	1	0	1	0	1	1	0	0	1
VNov	Bucbuc	1	0	1	0	1	0	1	0	1	0	0
VNov	Bursim	1	0	1	0	1	0	1	0	0	0	0
VNov	Capind	0	0	0	0	1	0	0	1	0	0	0
VNov	Casgau	1	0	1	0	0	0	0	1	0	0	0
VNov	Cecpel	1	0	0	0	0	0	1	0	0	0	0
VNov	Cocref	1	0	0	0	1	0	1	0	0	0	1
VNov	Croarb	1	0	0	0	1	0	0	0	0	0	0
VNov	Denarb	1	0	1	0	1	0	0	1	0	0	0
VNov	Drylat	1	0	1	0	1	0	1	0	0	0	1
VNov	Hamtri	0	0	0	0	0	0	0	0	0	0	0
VNov	Jatgau	1	0	0	1	1	0	0	1	0	0	0

VNov	Loncas	1	0	0	0	1	0	0	1	0	0	0
VNov	Longua	1	0	1	1	1	0	1	1	0	0	0
VNov	Manzap	1	0	1	1	1	1	1	0	0	0	0
VNov	Matopp	1	0	0	1	0	0	0	0	0	0	0
VNov	Metbro	1	0	1	0	1	0	1	1	0	0	1
VNov	Mosdep	1	0	0	0	0	0	1	1	0	0	1
VNov	Necsal	1	0	1	0	1	0	1	0	0	0	0
VNov	Pispis	1	0	1	0	1	0	0	1	0	0	0
VNov	Poucam	1	0	0	0	1	0	0	1	0	0	0
VNov	Procop	1	0	0	0	1	0	0	0	0	0	0
VNov	Ranlon	1	0	0	0	0	0	1	0	0	0	0
VNov	Simsal	1	0	0	0	1	0	0	0	0	0	0
VNov	Vitgau	1	0	0	0	1	0	0	0	0	0	0

Acronym of the species: *Acacia centralis* (Acacen), *Acacia cornigera* (Acacor), *Acacia gaumeri* (Acagau), *Allophylus cominia* (Allcom), *Amyris elemifera* (Amyele), *Annona reticulata* (Annret), *Aspidosperma desmanthum* (Aspdes), *Astronium graveolens* (Astgra), *Bauhinia divaricate* (Baudiv), *Bravaisia berlandieriana* (Braber), *Brosimum alicastrum* (Broali), *Bucida buceras* (Bucbuc), *Bursera simaruba* (Bursim), *Caesalpinia mollis* (*Caemol*), *Capparis indica* (Capind), *Cascabela gaumeri* (Casgau), *Cecropia peltate* (*Cecpel*), *Celtis trinervia* (*Celtri*), *Coccoloba cozumelensis* (Coccoz), *Coccoloba reflexiflora* (Cocref), *Coccoloba spicata* (Cocspi), *Cordia alliodora* (Corall), *Cosmocalyx spectabilis* (Cosspe), *Croton arboreus* (Croarb), *Croton icche* (Croicc), *Croton peraeruginosus* (Croper), *Cupania belizensis* (Cupbel), *Dendropanax arboreus* (Denarb), *Diospyros salicifolia* (Diosal), *Diospyros yatesiana* (Dioyat), *Drypetes lateriflora* (Drylat), *Erythroxylum rotundifolium* (Eryrot), *Esenbeckia berlandieri* (Eseber), *Eugenia ibarrae* (Eugiba), *Eugenia winzerlingii* (Eugwin), *Exostema caribaeum* (Exocar), *Exothea diphylla* (Exodip), *Guaiacum sanctum* (Guasan), *Guettarda combsii* (Guecom), *Guettarda gaumeri* (Guegau), *Gymnopodium floribundum* (Gymflo), *Hampea trilobata* (Hamtri), *Handroanthus chrysanthus* (Hanchr), *Jatropha gaumeri* (Jatgau), *Krugiodendron ferreum* (Krufer), *Licaria coriacea* (Liccor), *Lonchocarpus castilloi* (Loncas), *Lonchocarpus guatemalensis* (Longua), *Lonchocarpus rugosus* (Lonrug), *Lonchocarpus yucatanensis* (Lonyuc), *Lysiloma latisiliquum* (Lyslat), *Manilkara zapota* (Manzap), *Matayba oppositifolia* (Matopp), *Melicoccus oliviformis* (Meloli), *Metopium brownie* (Metbro), *Mosannona depressa* (Mosdep), *Myrciaria floribunda* (Myrflo), *Nectandra salicifolia* (Necsal), *Neea choriophylla* (Neecho), *Neomillspaughia emarginata* (Neoema), *Pimenta dioica* (Pimdio), *Piscidia piscipula* (Pispis), *Pouteria amygdalina* (Pouamy), *Pouteria campechiana* (Poucam), *Pouteria reticulata* (Pouret), *Protium copal* (Procop), *Pseudobombax ellipticum* (Pseell), *Pseudolmedia spuria* (Psespu), *Randia longiloba* (Ranlon), *Sebastiania adenophora* (Sebade),

Sideroxylon floribundum (Sidflo), *Sideroxylon obtusifolium* (Sidobt), *Sideroxylon salicifolium* (Sidsal), *Simarouba amara* (Simama), *Simira salvadorensis* (Simsal), *Spondias mombin* (Spomom), *Swartzia cubensis* (Swacub), *Thouinia paucidentata* (Thopau), *Trichilia glabra* (Trigla), *Trichilia havanensis* (Trihav), *Trichilia minutiflora* (Trimin), *Trophis racemosa* (Trorac), *Vitex gaumeri* (*Vitgau*), *Zuelania guidonia* (Zuegui).
