# Supplementary figures and images for: Linking Anthropogenic Landscape Perturbation to Herbivory and Pathogen Leaf Damage in Tropical Tree Communities
Source: Plants (Basel). 2023 Nov 13;12(22):3839. doi: 10.3390/plants12223839 (PMC10675074; doi:10.3390/plants12223839)

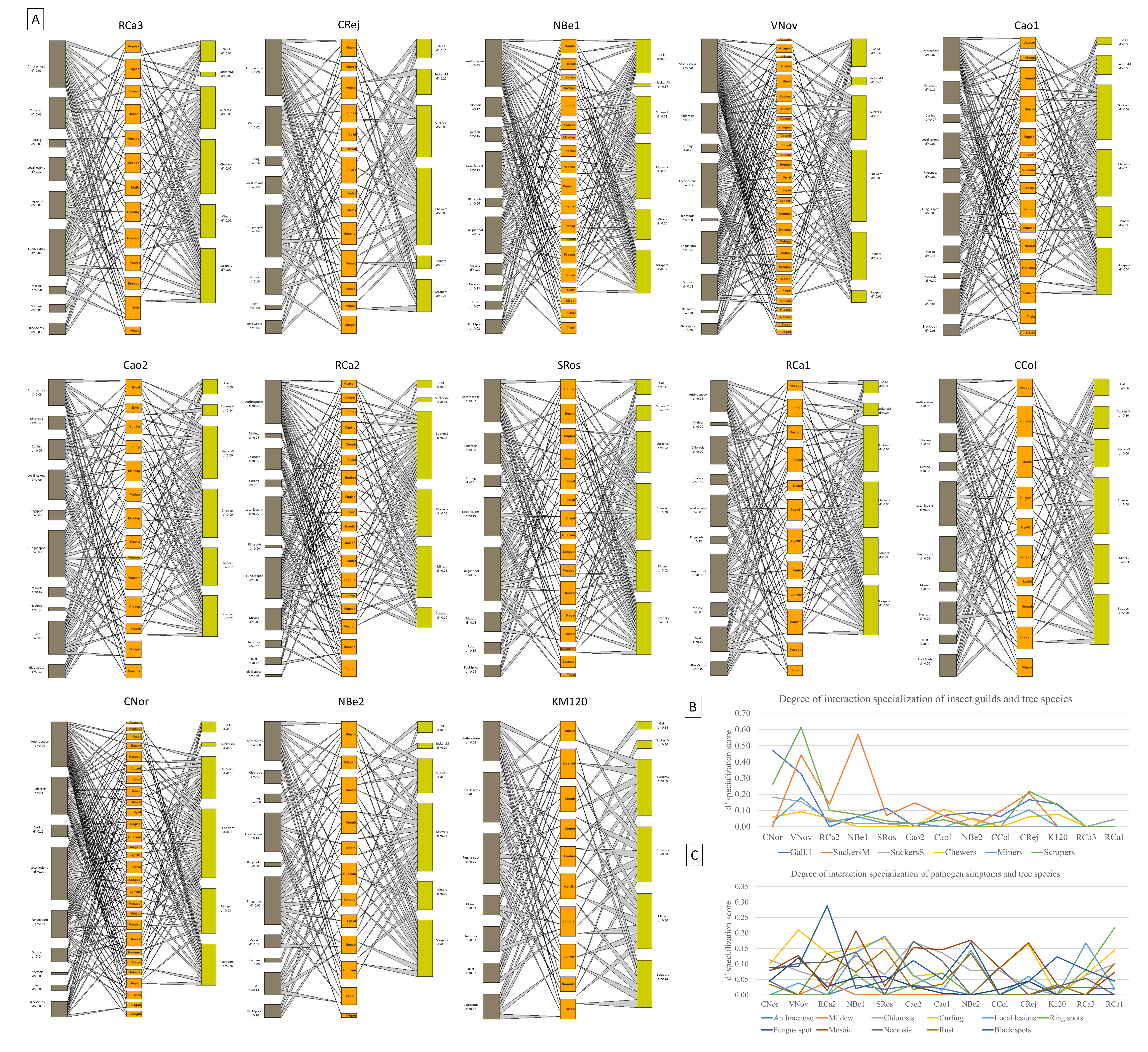

Supplement: Supplementary file 1 [file plants-12-03839-s001.zip › Fig. S1 Interaction networks between tree species, herbivorous and symptoms.tif]
